# GWAS analysis in spring barley (*Hordeum vulgare L*.) for morphological traits exposed to drought

**DOI:** 10.1371/journal.pone.0204952

**Published:** 2018-09-27

**Authors:** Mitra jabbari, Barat Ali Fakheri, Reza Aghnoum, Nafiseh Mahdi Nezhad, Reza Ataei

**Affiliations:** 1 Department of Plant Breeding and Biotechnology, Faculty of Agriculture, University of Zabol, Zabol, Sistan and Baluchestan province, Iran; 2 Seed and Plant Improvement Research Department, Khorasan Razavi Agricultural and Natural Resources Research and Education Center, AREEO, Mashhad, Iran; 3 Seed and Plant Improvement Institute, Agricultural Research, Education and Extension Organization (AREEO), Karaj, Iran; Huazhong University of Science and Technology, CHINA

## Abstract

Association analysis based on linkage disequilibrium has become a common and powerful approach for detection of QTLs underlying complex agronomic traits including drought tolerance. To determine marker/trait association, 148 modern European spring barley cultivars were evaluated under drought stress. Associations of morphological traits with AFLP/SSR markers were investigated based on the mixed linear model using the TASSEL_3.0_. Population structure was estimated using various methods including Bayesian clustering model by STRUCTURE software, PCoA analysis, NJ dendrogram and Hierarchical Clustering. Linkage disequilibrium patterns were explored among the whole genome and each chromosome separately. All the analysis for population structure divided the population into two sub-groups. Linkage disequilibrium analysis showed that by increasing genetic distance, LD decreases. Totally, 167 significant marker trait associations were found which delineated into 65 QTLs in both treatments. Two stable QTLs on 5H at 86.880 cM were detected for Internode Length and on 3H at 126.421 cM for flag leaf length in drought stress treatment. Fourteen QTLs were co-localized with previously reported QTLs and others were novel. The results indicate that these putative genomic regions contain genes that have pleiotropic effects on morphological traits in drought condition.

## Introduction

Drought is one of the climate change consequences affecting stable food production. In the last decade, temperature increases expected for the dry lands are in the range of 2–4°C, with a tendency in the tropical dry lands towards the lower part of the range, and in the non-tropical dry lands towards the higher part of the range [1–3]. Thus, drylands are the most severely affected by climate change. In most global change scenarios, water scarcity is a major determinant on agricultural land [4–6]. The development of drought-tolerant genotypes as well as genotypes with higher water-use efficiency is a global interest as populations continue to increase and water availability decreases [7,8]. Several morphological and physiological traits in barley contribute to drought tolerance [9,10] which indicates the interactions of the environment and the genotype [11]. Understanding the genetic basis of important traits under stress conditions can improve breeding approach.

To determine the genetic basis of traits, important genetic and genomic resources were developed in a wide range of barley species [12]. Microsatellite (SSR) markers occur at thousands of locations within an organism's genome [13], and in barley, they offer the potential for generating high-density genetic maps [14,15]. Plant geneticists have proposed the use of microsatellites for marker assisted selection (MAS) of desirable traits in plant breeding [16].

Recently, association analysis based on the evaluation of unrelated accessions provided an additional option to identify the loci (genes and/or QTLs) for traits [17–21]. In order to identify markers with allelic differences in significant levels, linkage disequilibrium (LD) was used in association mapping [22,23]. LD contents in a graphical format can be presented as LD plot of *D'* or *r*^*2*^ [24,25]. Genome-wide association mapping use to detect marker-trait associations for various traits in the whole genome [26–28]. Limitation of association mapping is spurious associations resulting from geographical origins and breeding history of genotypes in the panel named population structure. Several statistical methods have been designed to evaluate population structure, these include: a) Structured Association (SA) [29], b) Genomic Control (GC) [30], c) Mixed Model approach [31] and d) Principal Component approach [32].

The most published studies on association analysis in plants are on resistance to biotic stresses and quality traits, with a genetic basis less complex than abiotic stresses. Investigation of traits providing drought tolerance in maize [33,34] and durum wheat [27,35] has been reported. Several factors limit obtaining reliable QTLs and marker-assisted selection (MAS) in breeding programs. One of them is environmental dependence of QTL expression. Because of phenotypic variance and the direction of the additive effects of stress tolerance traits that is greatly influenced by environmental factors and the intensity of the stress, the effect of the same QTL can differ according to the environmental conditions [36].

In the previous studies on QTL mapping, several QTLs for important agronomic traits have been identified on different chromosomes in barley [12,37–43]. For an efficient marker assisted selection, understanding the interactions between environment and marker/trait QTLs are necessary, especially for complex traits under stress conditions [44–46,28]. In order to develop climate resilient crops, a better understanding of drought tolerance in them from molecular, phenological, morphological and physiological perspectives is required. Finding stable QTLs for drought tolerance traits, based on phonological stage exposed to drought stress is very important to determine the tolerant stage of growth in barley. Thus, this study was carried out to identify the population structure in a collection of 148 germplasm cultivars and the association of AFLP and SSR markers with morphological traits in barley exposed to drought stress.

## Materials and methods

### Plant material and experimental design

A total of 148 barley cultivars (S1 File), which are commercial cultivars in Western European countries [47], were evaluated under drought stress conditions. The seeds of association panel were received from Khorasan Razavi Agricultural and Natural Resources Research and Education Center. The experiment was conducted at Zahak Agricultural Research Station, Sistan and Baluchistan, Iran (Latitude = 30°15'N, Longitude = 60°15'E, Altitude = 480 m). Genotypes were evaluated using alpha-lattice design with two replications in two conditions: a. well-watered treatment (irrigation at 90% fc) and b. drought stress treatment (irrigation at 40% fc) during 2015–2016 and 2016–2017. Each replicate contains 11 incomplete blocks with 14 plots, and each replication included 148 barley cultivars and six local barley varieties (Local, 5- White cluster salinity, Nomar, Zahak, NP-90-113 and Nimroz) to complete the blocks. Each germplasm accession was sown in four-row planes with the length of 2 m and 20 cm distance between lines. After initial irrigation for germination, subsequent irrigations for well-watered treatment were done after the soil moisture reached 90% of the field capacity, and for dryness, treatment were after reaching 40%. The measurement was done using Time-Domain Reflectometer (TDR) method and during the growing season, the necessary crop managements such as fertilizer use, weeding, pest and disease control were carried out. Data were recorded from ten plants randomly.

Morphological traits including the average number of tillers per plant (ANTP), plant height (PH), main spike length (MSL), internode length (IL), flag leaf length (FLL), flag leaf width (FLW), peduncle length (PL), flag leaf sheath length (FLSL), awn length (AL), number of main stem nodes (MSN) and grains per spike (GRS) were measured for two years in both irrigation conditions.

Variance components of phenotypic data were estimated using GenStat (15th edition) [48]. Heritabilities (Family Mean basis) were calculated using SAS_9.0_ software and SPSS_24_ was used to estimate the correlation of all the traits. Best Linear Unbiased Estimates (BLUEs) of phenotypic data based on G×E variances were used in association analysis [49].

### Genotypic analysis and population structure

The population was already genotyped by Kraakman et al. [50] using 14 AFLP primer combinations and 11 SSRs [47,50]. Aghnoum et al. [51] (unpublished data) added 21 new SSR marker to the previously reported map. Totally, with consideration of different alleles of SSRs, 407 polymorphic markers were used in the present study. In order to analyse genotyping data, markers with missing data of more than 10% and the frequency of minor alleles fewer than 5% were eliminated, and a new set of 218 markers was used to evaluate population structure and association analysis. The content of the polymorphic information (PIC) was calculated for each marker using Powermarker_3.25_ [52,53].

In order to estimate the population structure, different methods and different software packages were used and compared. At first, Bayesian clustering model [54] was performed using STRUCTURE_2.3_ and STRUCTURE HARVESTER software with a burn-in of 100 000 iterations and 100 000 Markov Chain Monte Carlo (MCMC) iterations using the admixture model. ΔK index was calculated to determine the most probable number of sub-populations and for the best K value, Q-matrix was extracted [29,47,38]. In the second step, principal coordinate analysis (PCoA) was performed based on the dissimilarity matrix using PAST_3_ software [38]. In the third approach, Neighbor-Joining dendrogram (NJ) was constructed based on genetic distance matrix using Tassel_5_. In order to compare several methods of estimation, the sub-groups in a panel, hierarchical cluster analyses based on UPGMA algorithm were also performed using Past_3_.

### Linkage disequilibrium and association analysis

The resolution of mapping the trait and the number of markers required for AM is affected by LD extent and the population structure has an effect on LD [38]. Linkage disequilibrium estimation was performed on all the panels and each chromosome separately by using squared allele frequency correlations (r^2^) between the pairs of loci with Haploview_4.01_ software [55].

Association analysis based on the mixed linear model (MLM) with Kinship-matrix and Q-matrix was performed using TASSEL_3_ [56]. A kinship matrix is a pair-wise relationship matrix which was calculated with all markers using TASSEL_3_ [57]. Mixed linear model (MLM) in comparison with other models for detecting marker/trait associations such as the general linear model (GLM), has the ability to reduce the false-positive associations with controlling both types I and II errors [38,28]. For assessment of the significant marker trait associations, threshold P-value of 0.03 was considered for all traits according to the approach proposed by Chan et al. [58] and Pasam et al. [38].

## Results

### Phenotypic data

All traits and environments showed highly significant differences. The variance analysis confirmed high phenotypic variability, which revealed that all traits were severely influenced by environmental factors, showing significant genotype (G), the interaction between genotype and year (G×Y), and the interaction between genotype, environment and year (G×E×Y). Genotype and environment interactions were also significant (P < 0.01) for all the traits except PH (S2 File). Over the well-watered treatment GRS showed high heritability (0.89), moderately heritabilities were observed for PH, MSL, AL and FLW with the values between 0.60–0.69, whereas ANTP and FLL showed poorly heritabilities: 0.37 and 0.11 respectively. In drought treatment all traits showed moderately heritable with a values between 0.43–0.68, whereas GRS showed high heritability (0.76). The statistics and heritabilities of the traits are presented in Table 1.

**Table 1:** Estimation of mean, minimum (Min), maximum (Max) and heritabilities (h^2^) of all the traits based on data from well-watered (W) and drought stress treatments (D) across two years.

Trait	environment	year	Min	Max	Mean	Broad sense heritability—h^2^ (%)
ANTP	W	2015	1.68	4.01	2.85	0.37±0.20
		2016	1.18	3.51	2.35	
	D	2015	1.95	5.05	2.90	0.12±0.09
		2016	1.80	4.00	2.71	
PH	W	2015	55.99	115.5	92.95	0.60±0.12
		2016	43.50	96.40	71.57	
	D	2015	43.5	108.2	69.77	0.68±0.086
		2016	47.3	92.1	63.61	
IL	W	2015	10.68	19.24	14.72	0.32±0.22
		2016	7.19	17.97	12.20	
	D	2015	6.60	17.07	11.39	0.43±0.17
		2016	6.01	15.03	10.83	
MSL	W	2015	4.62	11.53	8.18	0.68±0.083
		2016	4.18	12.40	7.95	
	D	2015	3.30	16.30	7.42	0.46±0.16
		2016	3.5	12.2	7.64	
AL	W	2015	13.18	25.41	8.18	0.60±0.11
		2016	13.75	25.19	20.50	
	D	2015	10.2	22.6	16.51	0.56±0.12
		2016	12.3	25.3	18.40	
FLSL	W	2015	14.61	27.57	21.68	0.52±0.14
		2016	14.98	25.98	20.50	
	D	2015	11.6	25.46	18.37	0.68±0.083
		2016	13.6	25.4	19.49	
PL	W	2015	10.59	32.15	20.83	0.52±0.14
		2016	16.24	28.34	2.78	
	D	2015	6.99	29.43	15.41	0.56±0.12
		2016	10.3	36.5	19.81	
FLL	W	2015	3.63	22.79	11.33	0.11±0.36
		2016	3.23	11.49	6.61	
	D	2015	2.37	20.98	10.58	0.54±0.13
		2016	3.02	11.9	6.49	
FLW	W	2015	0.31	1.86	0.80	0.69±0.078
		2016	0.22	1.27	0.63	
	D	2015	0.22	1.51	0.77	0.58±0.11
		2016	0.30	1.08	0.59	
MSN	W	2015	2.48	6.97	4.63	0.50±0.15
		2016	3.00	6.29	4.43	
	D	2015	3.21	6.82	4.47	0.50±0.15
		2016	3.00	6.00	4.22	
GRS	W	2015	15.77	67.42	28.30	0.89±0.021
		2016	15.34	61.47	25.37	
	D	2015	12.51	57.61	22.47	0.76±0.053
		2016	14.5	45.03	22.76	

The correlation coefficient between all traits is shown in Table 2. PH was found to be positively correlated with other traits except MSL in well-watered treatment, but it showed weak negative correlation with ANTP. In drought treatment, it has no significant correlation with ANTP, MSL and FLW but a significant positive correlation with other traits. A negative correlation was observed between ANTP and MSL with GRS in both treatments. Correlations among FLSL and MSN were also negatively significant in both treatments. The traits FLL and FLW were highly correlated in both treatments, and GRS was shown to be strongly correlated with both. Three morphological traits: IL, AL and PL also had significant positive correlation with each other in both treatments. Significant negative correlation was detected between IL and MSN with the correlation coefficients of -0.277 and -0.297 in drought and well-watered treatments, respectively. In drought treatment, ANTP was not significantly correlated with other traits except GRS.

**Table 2:** Correlation coefficients among different traits based on data from each treatment for the two years.

Trait	ANTP	PH	IL	MSL	AL	FLSL	PL	FLL	FLW	MSN	GRS
ANTP		-0.114	-0.009	-0.133	-0.141	-0.079	-0.070	-0.153	-0.153	-0.030	-0.166*
PH	-.0223*		0.558**	0.127	0.374**	0.539**	0.636**	0.209**	0.126	0.262**	0.464**
IL	-0.061	0.273**		0.164*	0.217**	0.531**	0.597**	0.131	0.019	-0.277**	0.115
MSL	0.009	0.032	0.219**		0.555**	0.301**	0.034	0.041	-0.052	-0.127	-0.284**
AL	-0.024	0.261**	0.212**	0.586**		0.593**	0.263**	0.149	-0.017	0.195*	-0.062
FLSL	-0.159*	0.287**	0.400**	0.237**	0.455**		0.660**	0.124	-0.135	-0.288**	0.169*
PL	-0.205*	0.673**	0.407**	-0.068	0.191*	0.476**		0.303**	0.190*	-0.190*	0.377**
FLL	-0.089	0.406**	0.133	0.054	0.095	-0.014	0.439**		0.658**	-0.098	0.291**
FLW	-0.211**	0.413**	0.059	0.130	-0.056	-0.086	0.415**	0.698**		0.038	0.356**
MSN	-0.113	0.347**	-0.297**	-0.119	-0.118	-0.237**	-0.043	-0.016	0.141		0.351**
GRS	-0.262**	0.573**	0.010	-0.352**	-0.086	0.065	0.517**	0.406**	0.631**	0.316**	

*, **: Significant at 0.05 and 0.01 level, respectively.

Values above the diagonal are correlation coefficients in drought treatment; values below the diagonal are correlation coefficients in well-watered treatment.

### Genetic diversity and population structure

**Fig 1:**
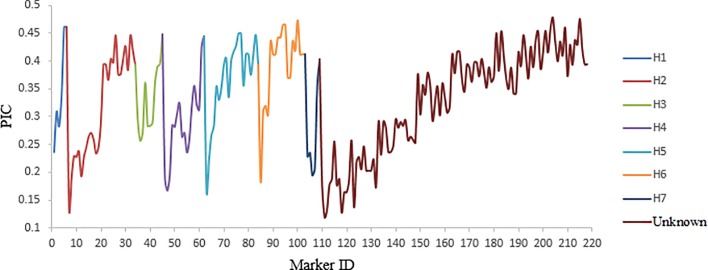
Polymorphic information content of 218 markers in the panel.

From 407 AFLP and SSR markers, 189 markers with missing data more than 10% and MAF below 5% were excluded. Therefore, 218 markers were considered for this study. The range of PIC value for markers was from 0.12 to 0.47 with an average of 0.33, indicating the informativeness of markers in the current study. Polymorphic information contents for most markers were higher than 0.25 (Fig 1). The average PIC index on each chromosome was from 0.27 on 7H to 0.40 on 6H.

**Fig 2:**
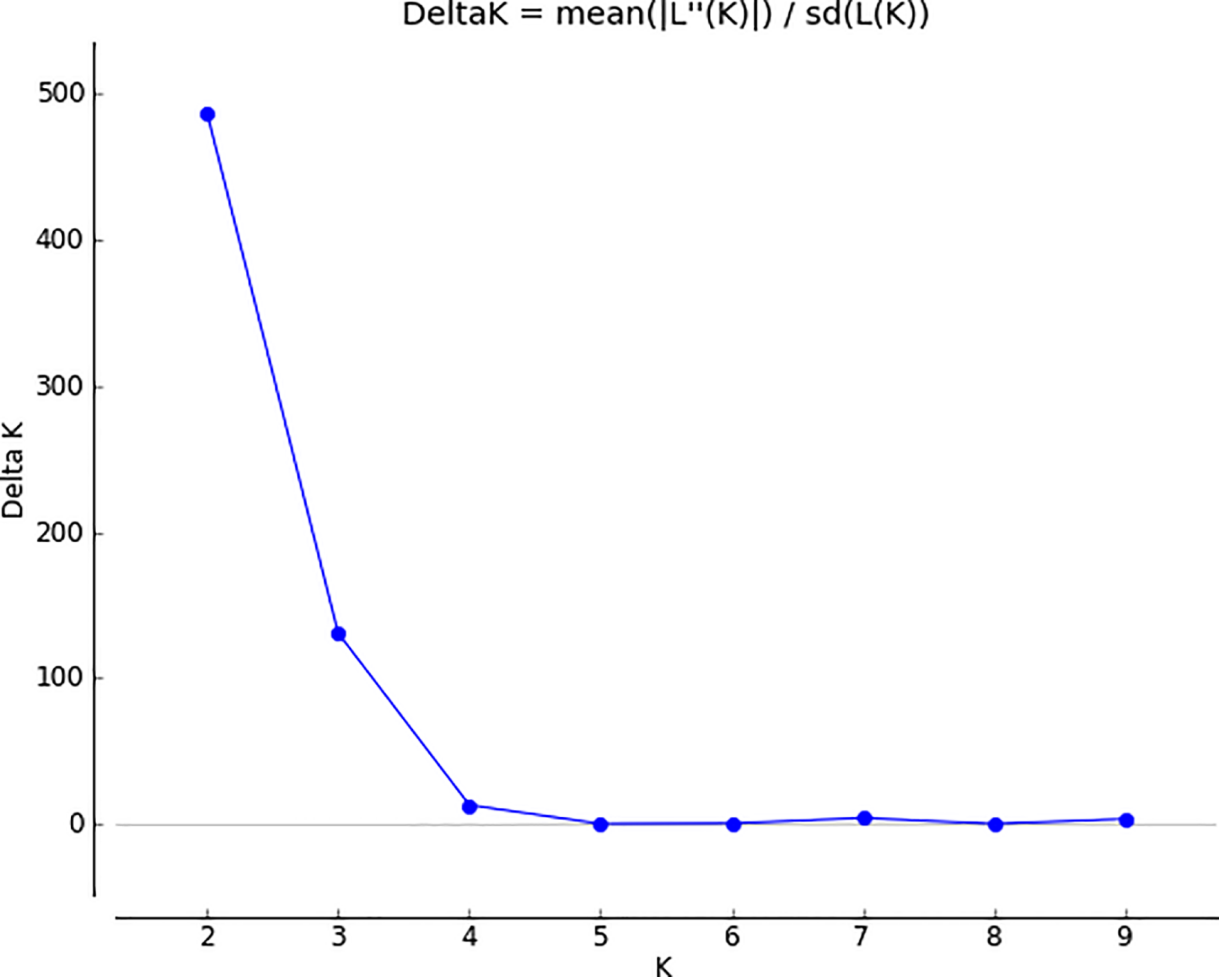
Structure Harvester results using 218 markers.

The existence of sub-populations in 148 barley cultivars using 218 markers was investigated using the Bayesian method, and the number of subgroups was determined using Structure software2.3 [59]. The population structure matrix (Q) was defined by ΔK index, highest value was obtained at K = 2, which indicates that there are two sub-groups in this population (Fig 2).

The vertical axis represents the value of ΔK statistic and the horizontal axis showed the number of clusters [60]. The two groups are defined as: K1: 83 barley cultivars and K2: 67 barley cultivars. The separation of these groups cannot be explained by morphological or geographical differences between cultivars [47].

**Fig 3:**
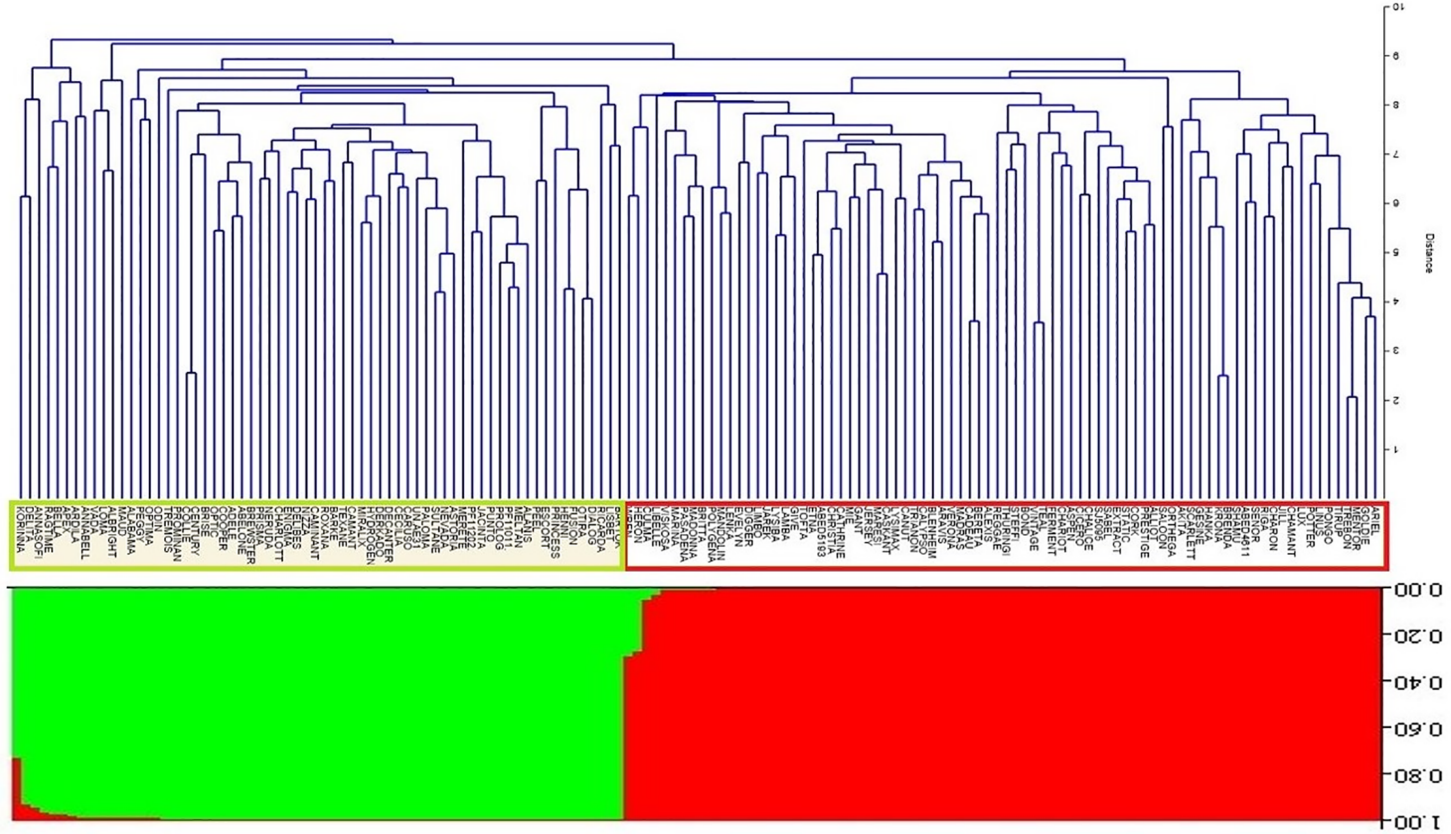
The comparison of UPGMA clustering of the accessions based on their genetic distances and the bar plot result according to the structure analysis, both dividing the panel into 2 distinct sub-groups K1 and K2.

The distributions of all the accessions within the two groups based on the relatively genetic distances were compared using structure and cluster analysis (Fig 3). In the bar plot diagram, each individual is represented in K colored segments with lengths proportional to each of the K1 or K2 subgroups. The part below the diagram represents the hierarchical clustering (UPGMA) dendrogram of all the accessions, once again, two completely separate clusters were identified, which correspond well with genetic distances.

**Fig 4:**
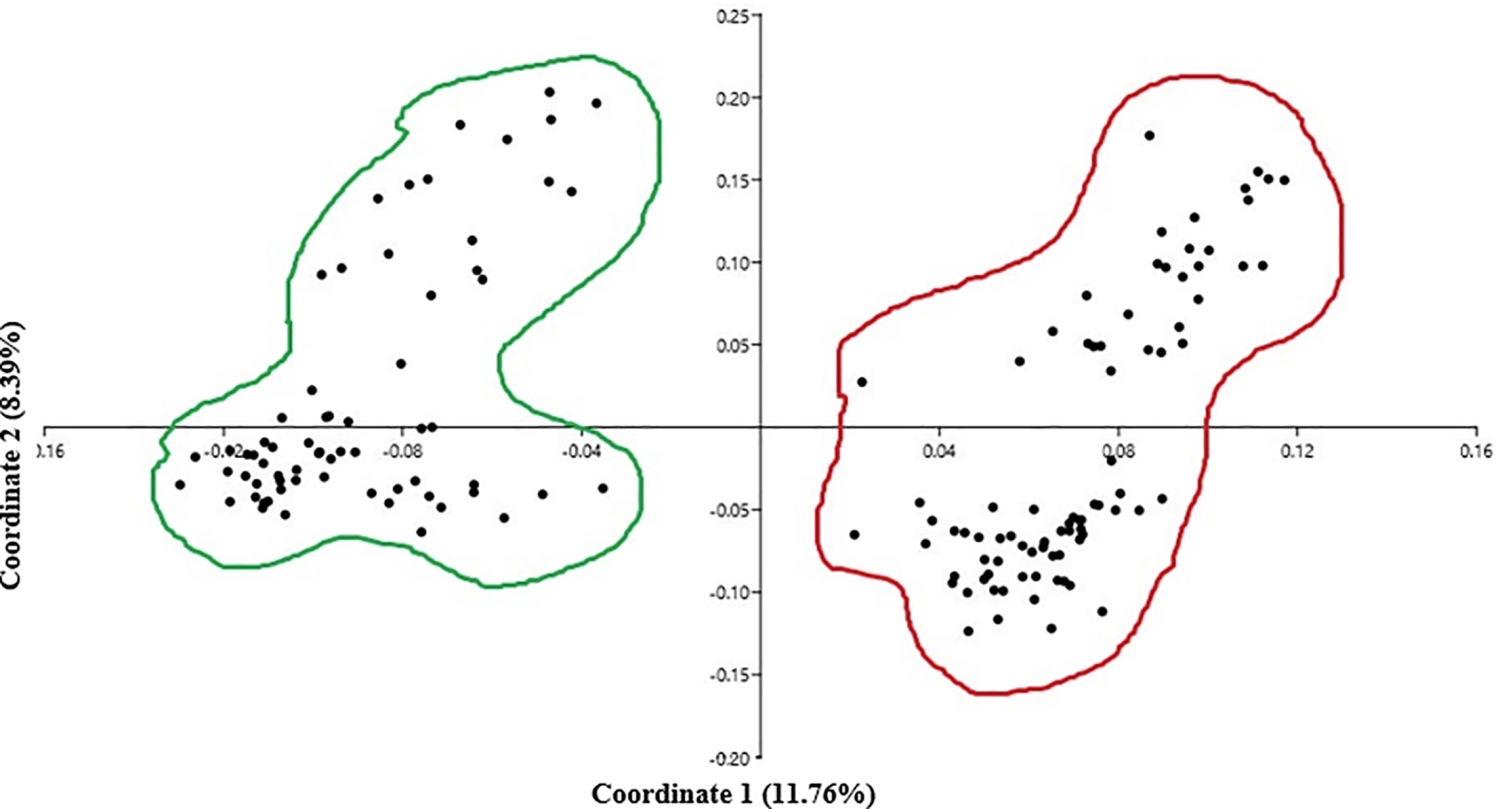
PCoA analysis of 148 barley cultivars and division of the population into two distinct sub-groups.

The dominant structure of the population was confirmed by PCoA (Fig 4) and NJ dendrogram (not shown). The PCoA analysis was carried out on a similarity matrix. Scatter plots showed distinct clustering identifying two main subgroups. In the PCoA, it is obvious that the first and second coordinates accounted for 11.76 and 8.39% of the genetic variation, respectively. Without reservation, identification the population structure was consistent using various methods, and the total molecular diversity was explained by diversity between and within these groups.

### Linkage disequilibrium

**Fig 5:**
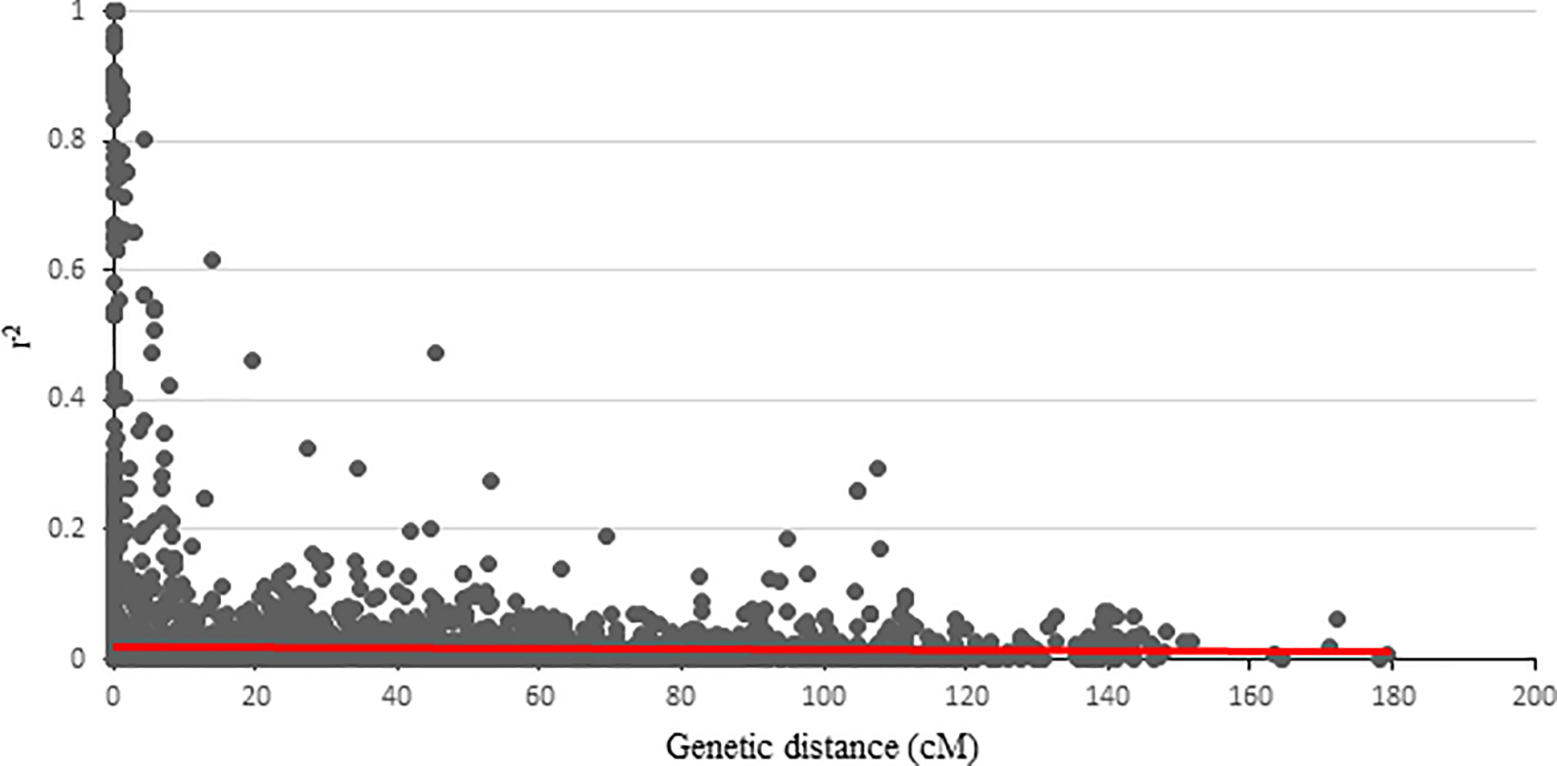
LD decay of marker pairs by increasing genetic distance (cM). The horizontal line indicates the 90th percentile distribution of unlinked r^2^.

Association mapping is a method in which QTL identification is based on linkage disequilibrium. Linkage disequilibrium analysis was carried out using 218 markers for all the panel and each chromosome separately using squared-allele frequency correlations (r^2^). The scatter plot showed that LD values decay with the genetic distance in population (Fig 5). A critical value of r^2^ (90th percentile distribution) was estimated to be 0.0178, and the values above were assumed to be caused by genetic linkage. In the panel, it was observed that LD varied along each chromosome with regions of high and low LD (Table 3).

**Table 3:** The mean LD values on seven chromosomes of barley.

Chromosome	Map length (cM)	Mean r^2^		
		D* <5 cM	D = 5–10 cM	D > 10 cM
H1	102.49	0.971	0.027	0.0033
H2	154.422	0.191	0.111	0.021
H3	151.031	0.613	0.043	0.015
H4	125.075	0.0293	0.086	0.014
H5	184.79	0.0179	0.073	0.024
H6	121.819	0.860	0.017	0.017
H7	140.172	1	0.0076	0.022

*Genetic distance

The pairs of loci were classified into three groups based on genetic distances: < 5 cM (tightly linked markers), 5–10 cM (moderately linked markers) and > 10 cM (loosely linked markers) [26,38]. The highest r^2^ values among all loci pairs were observed for tightly linked markers (< 5 cM) in different chromosomes, which indicate the linkage is one of the most effective forces causing disequilibrium in the population. However, because of the other effective factors on linkage disequilibrium such as population structure, genetic drift, migration, selection and mutation, it did not disappear as the distance between the markers increased.

### Marker-trait associations

Association analysis of morphological traits with 218 AFLP and SSR markers for well-watered and drought stress conditions was implemented using mixed linear model (MLM) approach based on Q-matrix and K-matrix. Marker trait association was considered when the main effect of marker was significant at 0.03 (-log10 (0.03) = 1.5) [38] (Tables 4,5,6,7 and 8, S1 and S2 Figs). One of the most important traits in barley is average number of tiller per plant, which directly affected biological yield. In this study, seven markers were found to be significantly associated with ANTP; among these markers, five unknown associated markers and two QTLs located at regions 71.336 and 86.880 cM on 4H (W) and 5H (D), respectively, were observed (Table 4).

**Table 4:** GWAS results for traits average number of tillers per plant (ANTP), plant height (PH) and main spike length (MSL).

Traits	treatment	year	Marker	Linkage group	Position (cM)	-Log (P)	R^2^	QTL
ANTP	W	2016	E35M48-087	Unmapped	-	2.87	0.079	-
		2017	E37M33-153	4H	71.336	2.007	0.049	*W2Q1ANTP4H*
			E42M48-196	Unmapped	-	1.57	0.035	-
			E42M48-195	Unmapped	-	1.53	0.034	-
	D	2016	Bmag0223-163	5H	86.880	1.56	0.047	*D1Q1ANTP5H*
		2017	E37M33-151	Unmapped	-	2.05	0.061	-
			E37M33-152	Unmapped	-	1.81	0.052	-
PH	W	2016	E39M61-133	7H	125.104	2.05	0.055	*W1Q1PH7H*
		2017	E45M49-210	Unmapped	-	1.94	0.046	*-*
			E45M55-164	Unmapped	-	1.88	0.044	*-*
			E38M50-284	Unmapped	-	1.59	0.035	*-*
			E38M50-336	Unmapped	-	1.56	0.035	*-*
			E35M55-171	Unmapped	-	1.56	0.034	*-*
	D	2016	E38M50-119	Unmapped	-	1.88	0.053	*-*
			E35M54-163	Unmapped	-	1.79	0.049	*-*
			E38M50-456	Unmapped	-	1.53	0.040	*-*
		2017	Bmag0223-170	5H	86.880	2.26	0.066	*D2Q1PH5H*
			E37M33-226	Unmapped	-	2.01	0.056	*-*
			E35M54-163	Unmapped	-	1.95	0.052	*-*
			E42M32-228	Unmapped	-	1.65	0.043	*-*
MSL	W	2016	Bmag0223-170	5H	86.880	1.68	0.054	*W1Q1MSL5H*
			E42M32-184	5H	41.400	1.51	0.048	*W1Q2MSL5H*
		2017	-	-	-	-	-	*-*
	D	2016	E42M32-272	2H	14.767	2.35	0.061	*D1Q1MSL2H*
			E35M61-378	2H	3.800	2.02	0.052	*D1Q2MSL2H*
			E37M33-134	Unmapped	-	1.81	0.046	*-*
			E42M32-271	Unmapped	-	1.80	0.043	*-*
			E35M48-170	Unmapped	-	1.76	0.039	*-*
			E42M32-378	2H	106.551	1.65	0.038	*D1Q3MSL2H*
		2017	E38M54-112	Unmapped	-	1.97	0.047	*-*
			E35M61-182	6H	95.421	1.63	0.036	*D2Q1MSL6H*
			E35M55-407	1H	64.102	1.52	0.033	*D2Q2MSL1H*

W: well-watered treatment; D: drought stress treatment.

**Table 5:** GWAS results for traits internode length (IL) and flag leaf length (FLL).

Traits	treatment	year	Marker	Linkage group	Position (cM)	-Log (P)	R^2^	QTL
IL	W	2016	E42M48-203	5H	157.148	1.90	0.046	*W1Q1IL5H*
			HVM040-162	4H	15.800	1.74	0.042	*W1Q2IL4H*
			E38M55-219	Unmapped		1.62	0.036	*-*
			E33M54-095	Unmapped		1.62	0.036	*-*
			E45M49-069	Unmapped		1.52	0.032	*-*
		2017	E39M61-247	1H	95.263	2.23	0.054	*W2Q1IL1H*
			E45M49-285	Unmapped	-	2.21	0.055	*-*
			E38M50-336	Unmapped	-	1.93	0.045	*-*
			E35M54-243	2H	11.874	1.85	0.044	*W2Q2IL2H*
			E42M32-254	Unmapped	-	1.84	0.044	*-*
			E38M50-284	Unmapped	-	1.73	0.039	*-*
	D	2016	E38M50-456	Unmapped	-	1.62	0.038	*-*
			Bmag0223-170	5H	86.880	1.58	0.037	*D1Q1IL5H*
			E42M32-187	Unmapped	-	1.57	0.035	*-*
		2017	Bmag0223-170	5H	86.880	2.87	0.077	*D2Q1IL5H*
			E45M55-108	Unmapped	-	2.35	0.062	*-*
			E37M33-153	4H	71.336	2.01	0.048	*D2Q2IL4H*
			E33M54-421	Unmapped	-	1.86	0.043	*-*
			E35M54-163	Unmapped	-	1.85	0.042	*-*
			E33M54-095	Unmapped	-	1.70	0.038	*-*
			E37M33-501	2H	140.642	1.62	0.036	*D2Q3IL2H*
			E35M54-180	7H	140.172	1.53	0.033	*D2Q4IL7H*
			E33M54-055	Unmapped	-	1.52	0.032	*-*
			E33M54-230	2H	134.720	1.51	0.032	*D2Q5IL2H*
FLL	W	2016	E35M48-384	Unmapped	-	1.71	0.038	*-*
			E38M54-245	Unmapped	-	1.57	0.034	*-*
		2017	E35M61-378	2H	3.800	3.87	0.11	*W2Q1FLL2H*
			E38M55-114	5H	5.554	2.54	0.065	*W2Q2FLL5H*
			E42M48-203	5H	157.148	2.11	0.052	*W2Q3FLL5H*
			E35M48-087	Unmapped	-	1.72	0.038	*-*
			E38M50-120	Unmapped	-	1.56	0.035	*-*
			E42M48-380	6H	121.819	1.52	0.035	*W2Q4FLL6H*
	D	2016	E42M32-333	2H	117.122	2.70	0.079	*D1Q1FLL2H*
			E37M33-93	3H	126.421	1.56	0.037	*D1Q2FLL3H*
			HVM54-150	2H	122.406	1.52	0.034	*D1Q3FLL2H*
		2017	E42M32-273	Unmapped	-	2.69	0.072	*-*
			E38M54-127	Unmapped	-	2.01	0.047	*-*
			E42M32-271	Unmapped	-	1.94	0.046	*-*
			E35M48-170	Unmapped	-	1.85	0.042	*-*
			E35M61-182	6H	95.421	1.80	0.042	*D2Q1FLL6H*
			E45M49-382	Unmapped	-	1.75	0.041	*-*
			E45M49-380	Unmapped	-	1.67	0.038	*-*
			E37M33-93	3H	126.421	1.62	0.037	*D2Q2FLL3H*
			E38M55-139	4H	68.628	1.56	0.035	*D2Q3FLL4H*
			HVM54-150	2H	122.406	1.54	0.034	*D2Q4FLL2H*

W: well-watered treatment; D: drought stress treatment.

**Table 6:** GWAS results for traits flag leaf width (FLW) and peduncle length (PL).

Traits	treatment	year	Marker	Linkage group	Position (cM)	-Log (P)	R^2^	QTL
FLW	W	2016	E42M32-231	7H	25.313	1.63	0.037	*W1Q1FLW7H*
			E35M61-068	Unmapped	-	1.61	0.036	*-*
			E35M48-384	Unmapped	-	1.53	0.034	*-*
			E35M48-250	3H	10.668	1.52	0.033	*W1Q2FLW3H*
			E37M33-090	Unmapped	-	1.51	0.035	*-*
		2017	E35M61-378	2H	3.800	2.83	0.075	*W2Q1FLW2H*
			E35M48-087	Unmapped	-	2.15	0.050	*-*
			E35M48-170	Unmapped	-	1.66	0.038	*-*
			E42M32-250	5H	130.999	1.62	0.037	*W2Q2FLW5H*
			E38M50-385	Unmapped	-	1.52	0.035	*-*
	D	2016	E38M54-133	4H	125.075	1.89	0.054	*D1Q1FLW4H*
			E38M54-247	5H	12.543	1.87	0.052	*D1Q2FLW5H*
		2017	E42M32-273	Unmapped	-	1.98	0.051	*-*
			E38M55-219	Unmapped	-	1.89	0.044	*-*
			E37M33-218	Unmapped	-	1.73	0.042	*-*
			E37M33-152	Unmapped	-	1.67	0.039	*-*
			E37M33-151	Unmapped	-	1.63	0.038	*-*
			E42M32-271	Unmapped	-	1.60	0.037	*-*
PL	W	2016	E42M48-282	5H	114.402	1.65	0.038	*W1Q1PL5H*
			E35M48-087	Unmapped	-	1.51	0.032	*-*
		2017	E42M32-529	Unmapped	-	2.56	0.073	*-*
			E45M55-164	Unmapped	-	1.57	0.035	*-*
			E38M55-114	5H	5.554	1.54	0.034	*W2Q1PL5H*
			E45M55-108	Unmapped	-	1.52	0.035	*-*
	D	2016	E38M50-456	Unmapped	-	2.01	0.049	*-*
			E38M50-119	Unmapped	-	1.92	0.045	*-*
			E35M54-243	2H	11.874	1.74	0.039	*D1Q1PL2H*
			HVM54-150	2H	122.406	1.55	0.035	*D1Q2PL2H*
			HVM54-167	2H	122.406	1.53	0.035	*D1Q3PL2H*
		2017	E42M48-139	4H	63.099	1.95	0.054	*D2Q1PL4H*
			Bmac0134-132	2H	10.867	1.87	0.052	*D2Q2PL2H*
			E33M54-421	Unmapped	-	1.76	0.049	*-*
			E45M49-144	Unmapped	-	1.67	0.046	*-*
			E35M54-163	Unmapped	-	1.56	0.040	*-*

W: well-watered treatment; D: drought stress treatment.

**Table 7:** GWAS results for traits flag leaf sheath length (FLSL) and awn length (AL).

Traits	treatment	year	Marker	Linkage group	Position (cM)	-Log (P)	R^2^	QTL
FLSL	W	2016	E35M48-170	Unmapped	-	2.86	0.073	*-*
			Bmag0223-160	5H	86.880	2.02	0.051	*W1Q1FLSL5H*
			E35M48-400	5H	183.752	2.006	0.046	*W1Q2FLSL5H*
			E38M55-219	Unmapped	-	1.67	0.037	*-*
		2017	E42M32-529	Unmapped	-	2.52	0.067	*-*
			E45M55-164	Unmapped	-	2.21	0.054	*-*
			E38M55-128	5H	72.095	2.20	0.053	*W2Q1FLSL5H*
			E38M55-129	5H	72.095	2.20	0.053	*W2Q2FLSL5H*
			E45M55-108	Unmapped	-	2.01	0.052	*-*
			E38M50-119	Unmapped	-	1.99	0.050	*-*
	D	2016	E38M50-456	Unmapped	-	3.87	0.107	*-*
			E35M54-163	Unmapped	-	2.79	0.070	*-*
			E38M50-119	Unmapped	-	2.43	0.061	*-*
			Bmag0223-170	5H	86.880	2.18	0.053	*D1Q1FLSL5H*
			E35M54-243	2H	11.874	2.03	0.048	*D1Q2FLSL2H*
			E35M48-170	Unmapped	-	1.94	0.046	*-*
			E42M32-178	Unmapped	-	1.58	0.039	*-*
		2017	E33M54-421	Unmapped	-	2.09	0.060	*-*
AL	W	2016	E38M55-219	Unmapped	-	2.11	0.068	*-*
			E38M54-112	Unmapped	-	1.56	0.046	*-*
		2017	E35M48-380	Unmapped	-	1.79	0.046	*-*
			E38M55-139	4H	68.628	1.53	0.038	*W2Q1AL4H*
	D	2016	E38M50-456	Unmapped	-	2.19	0.058	*-*
			E35M48-170	Unmapped	-	1.86	0.043	*-*
			E38M50-119	Unmapped	-	1.75	0.044	*-*
			E42M32-200	5H	76.744	1.66	0.040	*D1Q1AL5H*
			E42M32-178	Unmapped	-	1.61	0.038	*-*
		2017	E33M54-095	Unmapped	-	2.38	0.060	*-*
			E38M50-135	Unmapped	-	1.78	0.047	*-*
			E37M33-226	Unmapped	-	1.78	0.042	*-*

W: well-watered treatment; D: drought stress treatment.

**Table 8:** GWAS results for traits number of main stem nodes (MSN) and grains per spike (GRS).

Traits	treatment	year	Marker	Linkage group	Position (cM)	-Log (P)	R^2^	QTL
MSN	W	2016	E42M32-251	Unmapped	-	2.11	0.053	*-*
			E38M50-414	Unmapped	-	2.09	0.053	*-*
			E35M55-302	4H	55.763	2.05	0.047	*W1Q1MSN4H*
			E42M32-250	5H	130.999	1.90	0.046	*W1Q2MSN5H*
			E35M54-078	Unmapped	-	1.76	0.039	*-*
			E38M54-390	2H	88.013	1.64	0.036	*W1Q3MSN2H*
			E35M55-160	Unmapped	-	1.51	0.032	*-*
		2017	E38M55-128	5H	72.059	2.29	0.058	*W2Q1MSN5H*
			E38M55-129	5H	72.292	2.29	0.058	*W2Q2MSN5H*
			E42M32-069	Unmapped	-	1.85	0.044	*-*
	D	2016	E37M33-191	4H	45.305	2.65	0.070	*D1Q1MSN4H*
			E42M32-250	5H	130.999	2.26	0.057	*D1Q2MSN5H*
			E35M54-078	Unmapped	-	2.12	0.050	*-*
			E45M49-382	Unmapped	-	2.04	0.048	*-*
			E37M33-189	4H	45.305	2.01	0.049	*D1Q3MSN4H*
			E45M49-380	Unmapped	-	1.97	0.046	*-*
		2017	E42M32-228	Unmapped	-	2.31	0.071	*-*
			E45M49-382	Unmapped	-	1.62	0.046	*-*
			E45M49-380	Unmapped	-	1.52	0.041	*-*
GRS	W	2016	-	-	-	-	-	*-*
		2017	E42M32-250	5H	130.999	2.04	0.071	*W2Q1GRS5H*
			E38M54-112	Unmapped	-	1.65	0.051	*-*
	D	2016	E42M32-228	Unmapped	-	2.33	0.062	*-*
			E42M32-250	5H	130.999	2.28	0.060	*D1Q1GRS5H*
			E37M33-93	3H	126.421	1.75	0.043	*D1Q2GRS3H*
			E45M55-164	Unmapped	-	1.59	0.035	*-*
			E38M50-149	Unmapped	-	1.54	0.036	*-*
		2017	Bmac0316-170	6H	44.900	1.64	0.042	*D2Q1GRS6H*

W: well-watered treatment; D: drought stress treatment.

Six markers displayed significant associations with plant height (PH) in well-watered treatment and seven markers in drought stress treatment. Except two markers on 7H (W) and 5H (D), others were unknown markers. Eleven significant markers were found for Main Spike Length (MSL) totally, and two of them were on 5H in well-watered treatment and the others in drought treatment were divided into four groups; three of them were on 2H, one QTL on 6H, one QTL on 1H and four of them were unknown (Table 4). Twenty four markers were found to be significantly associated with the trait, Internode Length (IL), in both treatments in the two years. In well-watered treatment, QTLs were found on 5H, 4H (2016) and 1H, 2H (2017), while in drought treatment, they were observed on 5H, 4H, 2H and 7H. Flag leaf length (FLL) was found to be associated with eight markers in well-watered treatment and thirteen markers in drought treatment. There were four unknown associated markers in W and six unknown markers in D condition (Table 5). In well-watered experiment, ten markers were found to be significantly associated with the trait, flag leaf width (FLW) and in stress experiment, eight markers were found. These markers were distributed on 2H, 3H, 5H, 4H and 7H (Table 6). Six markers were found to be significantly associated with the peduncle length (PL) in W condition, two of them were on 5H and others were unknown. Ten markers were found in stress condition, four of them on 2H, one on 4H and five markers were unknown. Eighteen markers displayed significant associations with Flag Leaf Sheath Length (FLSL). Ten markers were found in well-watered treatment which includes six unknown markers and four markers on 5H. Eight markers were found in stress condition, which includes six unknown markers and two on 5H and 2H. SSR marker *Bmag0223* on 5H was identified as a significant marker with FLSL in both experiments (well-watered and drought). Results showed that there were twelve significant markers associated with Awn Length (AL), while all of them were unknown markers except one on 4H (W) and one on 5H (D) (Table 7).

Total number of markers associated with number of main stem nodes (MSN) was nineteen, ten in W and nine in D experiment conditions. In well-watered condition, five markers were on 4H, 2H, 5H and others were unknown markers. In drought stress condition, three markers were on 4H and 5H, and six were unknown. Eight markers were found to be significantly associated with grains per spike (GRS). One marker on 5H at 130.999 cM was detected in both well-watered and drought treatments. Except unknown markers, others were distributed over 3H, 5H and 6H (Table 8).

## Discussion

In this study, whole genome association mapping was used in a spring barley population with 148 diverse genotypes for agronomic traits. 7 to 24 significant markers associated with each analysed trait were detected. The quality of the phenotypic data affected GWAS strongly [20]. The heritability values observed in our panel indicate that much of the phenotypic variation was environmental. Phenotypical means showed an extensive variation in the panel. Because of high genetic diversity within this population, phenotypic variations for all traits were high in both treatments. In this panel, the average PIC (0.33) observed was comparable to previous studies, and PIC values were different among chromosomes. The highest average PIC values were 0.40 for chromosome 6H, which corresponds with the results of study on a set of European barley cultivars [61]. Analyses of population structure were carried out using various approaches (STRUCTURE, PCoA, NJ-dendrogram and hierarchical cluster analysis based on UPGMA algorithm) and similar results (two subgroups) were shown, which cannot be defined by morphological or geographical differences of genotypes [47]. Two rowed barley is grown in Europe as malt producing material. Reduction of genetic diversity is the result of using a limited number of principal progenitors in breeding programs. So, distinct subpopulations were formed as seen in the present panel [62]. Several studies have previously reported LD pattern in various populations of barley using different markers (AFLP, SSR, DArT and SNP) [38,47,63,64]. In this panel of barley accessions, the whole genome LD decays below 0.0178 (critical value of r^2^). The extent of LD in the present panel for different chromosomes was observed with various patterns. Various levels of LD decay in whole genome and among different chromosomes in barley populations were previously reported [65,66]. Despite GWAS approach advantages, it may identify false positives associations due to population structure [67,68]. Previous studies tested various statistical models to correct the effect of population structure [32,69]. Two subpopulations were detected in our panel and in order to control the false-positive associations, linear models were used. According to previous studies on comparison of GWAS models, QK model was used because of its better performance than others [38,67,69].

In this study, a total of 167 significant associated markers for important morphological traits in barley were identified in both treatments. In well-watered treatment, 73 associated markers were identified, 29 QTLs were distributed on all chromosomes except 6H, and others (44 markers) were unknown. In drought stress treatment, 94 putative QTL regions were found, 36 QTLs were located on chromosomes 1H to 7H, and 58 QTLs were unknown.

For the trait ANTP, one associated unknown marker (*E35M48-087*) was found in well-watered treatment. This marker affected FLL, FLW and PL as significant putative QTL in the same treatment. Two markers were identified to be associated with ANTP and FLW in the drought treatment. The genomic region on 4H at 71.336 cM affects ANTP (W) and IL (D).

Most of the economically important traits in barley are inherited quantitatively, for instance, plant height. PH is strongly influenced by the environmental condition, particularly, drought [41]. Plant height is under polygenic control, and represents one of the most important agronomic traits for barley [70,71]. In order to reduce lodging, semi-dwarf and dwarf cultivars were developed. In each geographic region, different genes or alleles were deployed. In America and Australia, the *sdw1* dwarfing gene and in European, two-rowed germplasm in its allelic form, termed *denso*, is frequently seen [38]. In well-watered condition, QTL (*W1Q1PH7H*) was found for plant height on 7H at 125.104 cM, Wang et al. [70] found QTL “*QPh*.*NaTx-7H*” on 7H but at different position (80.0 cM). In drought stress condition, one associated SSR marker (*Bmag0223*) was identified on 5H at 86.880 cM named *D2Q1PH5H*. This region affected other traits like MSL(W), FLSL(D) and also IL(D). In this region, Fragile Stem1 gene (*fst1* (*fs*)) was identified [72] and it was previously reported as “*QTL12_PHT*” for plant height [38]. A stable significant associated marker (*E35M54-163*) for Plant Height was detected in drought stress treatment. For adaptation when a plant is exposed to drought stress, genomic regions that control plant height changed. In other words, different genes or alleles have been deployed in different environmental conditions. Two unknown markers were identified as significant associated markers for PH and IL in well-watered treatment. Three strategic traits in barley: PH, PL and FLSL, in well-watered treatment and also GRS in drought condition, were affected by the same putative QTL region. Plant height also had common associated markers with AL, MSN and GRS in drought treatment. These morphological traits are considered to be directly related to each other as the components of plant height.

Main Spike Length (MSL) is one of the yield components. Two QTLs were identified on 5H in well-watered treatment. Wang et al. [70] found QTL “*QSl*.*NaTx-5H*” on 5H for this trait but at different position (79.5 cM). In drought stress treatment, a significant genomic region was found on 2H at 3.800 cM, which is the same for FLL and FLW but ‘W’ treatment. QTL “*QSl*.*NaTx-2H*” was reported previously on 2H for MSL [70]. Another putative QTL on 6H at 95.421 was detected for MSL and FLL in ‘D’ treatment. Main Spike Length overlapped unknown associated markers with FLSL and AL in both treatments.

For the trait, Internode Length (IL), a significant QTL region was found on 5H at 157.148 in ‘W’ treatment, which is the same as FLL (W). On chromosome 2H at 11.874, a significant marker/trait association was identified for IL (W), PL (D) and FLSL (D). Also, a relationship between IL, PL and FLSL was observed by detecting same QTLs in both treatments. A stable QTL was detected on 5H (*Bmag0223*) for Internode Length in drought stress treatment.

Increase in grain yield is the most important aim of breeders; in order to achieve this goal, flag leaf effects has been widely studied [73–75]. In this study, for flag leaf length, two stable QTLs were identified on 3H at 126.421 cM and 2H at 122.406 cM in drought stress treatment. One stable QTL for FLL was reported on 2H, named”*qFLL2-12*” [74]. In well-watered treatment, a significant QTL on 5H at 5.554 cM was identified for FLL and PL. This region on 5H co-localized with previously mapped QTL “*AQDE022*” for Total Protein Content in barley [76]. Two characteristics of flag leaf (FLL and FLW) showed the same marker/trait association in ‘W’ and ‘D’ treatments. Flag leaf length in ‘D’ and awn length in ‘W’ treatment had the same QTL region on 4H at 68.628. QTL “*D2Q3FLL4H*” on 4H was detected for FLL and as previously reported, it affected QTL for Days to Heading [76].

Flag leaf width (FLW) is an important characteristic of flag leaf. For this trait in well-watered treatment, a putative QTL was identified on 3H at 10.668 cM. In the same region of 3H, QTL “*wst6* (*wst*.*j*)” was previously reported for the white streak 6 (white streak.j) trait [72]. A significant region on 5H as putative QTL for FLW, MSN and GRS was detected in ‘W’ treatment, also, for MSN and GRS in ‘D’ treatment. Several QTLs for this trait were found in both treatments on 2H, 3H, 4H, 5H and 7H. Gyenis et al. [73] detected three QTLs for flag leaf width on chromosomes 2H, 4H and 5H. Liu et al. [74] found one stable QTL (*qFLW4-18*) for FLW on 4H. QTL “*AQGZ036*” for Leaf Water Potential [76] was previously detected at the same region of 4H at 125.075 and QTL “*DSQ1FLW4H*” was found in drought treatment.

Peduncle length (PL) in well-watered treatment was affected by two putative regions on 5H, one of these at 5.554 cM was previously reported for Total Protein Content in barley [76]. A significant unknown marker associated with PL and FLSL was found in ‘W’ treatment. In drought treatment, three regions on 2H were detected to be associated with PL.

Flag Leaf Sheath Length (FLSL) had three QTLs on 5H in ‘W’ treatment, also, Pasam et al. [38] reported a QTL for plant height on 5H in barley. A significant marker on 5H was associated with two traits: FLSL and MSN in well-watered treatment. But in drought, FLSL and AL had the same associated marker. It seems this region has a pleiotropic effect on these traits.

Another component of yield is Awn length (AL): a putative QTL region on 4H was found in well-watered treatment, which was previously reported as Heading Days QTL [76]. While, genotypes were exposed to drought, a genomic region on 5H probably controlled Awn Length in barley. Wang et al. [70] found that QTL “*QAl*.*NaTx-5H*” on 5H for this trait near the QTL was at 75.8 cM. Awn Length has the same significant associated markers with PH, MSL, IL, FLSL and FLL in drought. Thus, it can be proved that such morphological traits have the same controller genomic regions in water deficiency. In ‘W’ treatment, AL, GRS, IL and FLSL were also affected by the same significant markers.

Number of main stem nodes (MSN) is a part of plant height in barley and has a direct effect on photosynthesis. In this study, 19 significant associated markers in both treatments were found for this trait. In well-watered treatment, a QTL region was detected on 2H at 88.013, which was previously reported as ‘*Zeo’* QTL for ‘*Zeocriton dwarf’* trait [77]. One stable QTL (unknown marker) was found for MSN in drought treatment. Another unknown marker was significant for this trait in both treatments. Number of main stem nodes in ‘W’ treatment has the same significant associated markers with FLSL, FLW and GRS. In drought condition, MSN, FLL, PH and GRS were also affected by the same significant markers.

Grains per spike (GRS) as one of the important yield components, has the major effect on final yield. According to significant associated markers found for the trait GRS, flag leaf characteristics (FLL, FLW and FLSL) have direct effect on GRS, especially when a plant is exposed to drought. A significant QTL on 3H was detected for this trait, which was found as stable QTL for FLL in drought stress.

In this study, several derived QTLs were congruent with previously identified QTLs in other mapping populations. Low R^2^ values (percentage of genetic trait variation explained) in the best associations for significant markers were observed. In many human studies, low R^2^ values have been reported and shown that the unexplained variation is due to unexplained missing heritability [78]. In plants GWAS studies, R^2^- values range from 0.2 to 3.95% as reported by Roy *et al*. [79], while in the present results, R^2^- values range from 3.20 to 11.0%. The “missing heritability” can be explained by insufficient marker coverage, rare alleles, large number of QTLs with small individual effects on a trait, and GWAS which is unable to detect epistatic interactions by these statistical approaches [79–81]. The above reasons were mainly discussed in human GWAS studies, but they also pertain to plants and other organisms studies. In addition, the statistical model used for analysis affects the variation explained by each marker. Detecting small effect markers will be reduced by increasing the threshold in the model [38].

## Conclusion

A large number of mapping populations have been developed to map QTL. Further, advanced mapping populations, including near-isogenic lines, chromosome segment substitution lines, and recombinant chromosome substitution lines [82–84], have been also developed to facilitate the genetic dissection of complex traits. As a result, many QTL controlling complex traits, including agronomic and morphological traits, yield component, disease resistance, tolerance to abiotic stress and malting quality, has been identified. Drought stress was applied after planting so that the effect of drought from germination can be screened, beginning from plant growth period.

Genotypic mechanism of drought-tolerant traits is complex, and the expression of stress-related genes should be identified in drought stress condition as compared to normal irrigation condition. QTL analysis in both conditions can improve quality related agronomical traits. Co-localizing of QTLs for two or more different traits was observed because of correlation between agronomical traits [39]. Comadran et al. [40] mentioned that co-localization of several QTL related to yielding components traits suggests that major developmental loci may be linked to most of the associations in barley.

Results of this study provide an insight into the genetic architecture of important morphological traits in barley. Fourteen QTLs were found to be co-localized with previously mapped QTLs for all the traits together, and others are novel QTLs. It was found that the number of QTLs for each trait in drought stress is more than that in well-watered treatment for the same trait. It suggests that genomic regions involved in response of plant to abiotic stresses are numerous, and are one of the reasons for barley adaptation to unfavorable environmental conditions. Detecting QTLs for some yield-related traits, such as main spike length, flag leaf length, flag leaf width and awn length in both experimental conditions can be used for improving high-yield resistance of barley genotypes to drought occurring at early stages of plant development.

These findings show that there is need to saturate the genetic map with additional types of markers to combine and overlap the current results with previously detected QTLs [85]. This will lead to the detection of supplemental associations and improve the significance of existing QTLs during water deficiency and would be useful in future marker-assisted barley breeding programs.

## Supporting information

S1 Fig Manhattan plots for GWAS on all traits in well-watered treatment for two years (PDF). (PDF)

S2 Fig Manhattan plots for GWAS on all traits in drought treatment for two years (PDF). (PDF)

S1 File List of spring barley cultivars and their breeders included in the study (PDF). (PDF)

S2 File Variance components for all traits of 148 barley cultivars evaluated in two environments. (PDF)
