# Supplementary material for: GWAS analysis in spring barley (Hordeum vulgare L.) for morphological traits exposed to drought
Source: PLoS One. 2018 Sep 27;13(9):e0204952. doi: 10.1371/journal.pone.0204952 (PMC6160164; doi:10.1371/journal.pone.0204952)
Supplement: S1 Fig — (PDF) [file pone.0204952.s001.pdf]

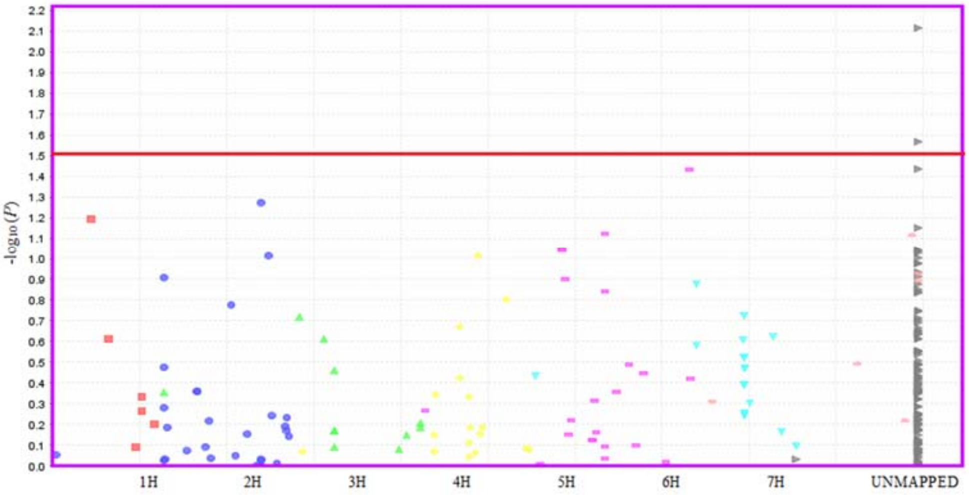

Manhattan Plot for AL in W1

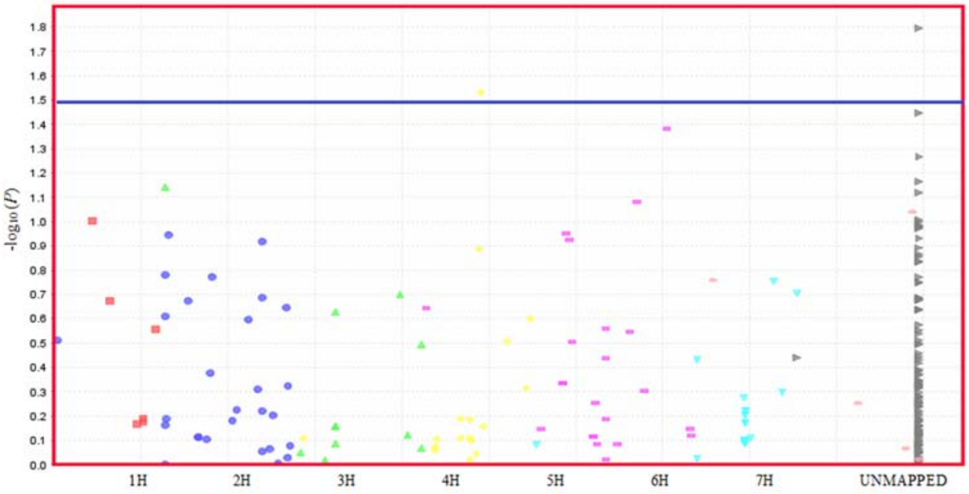

Manhattan Plot for AL in W2

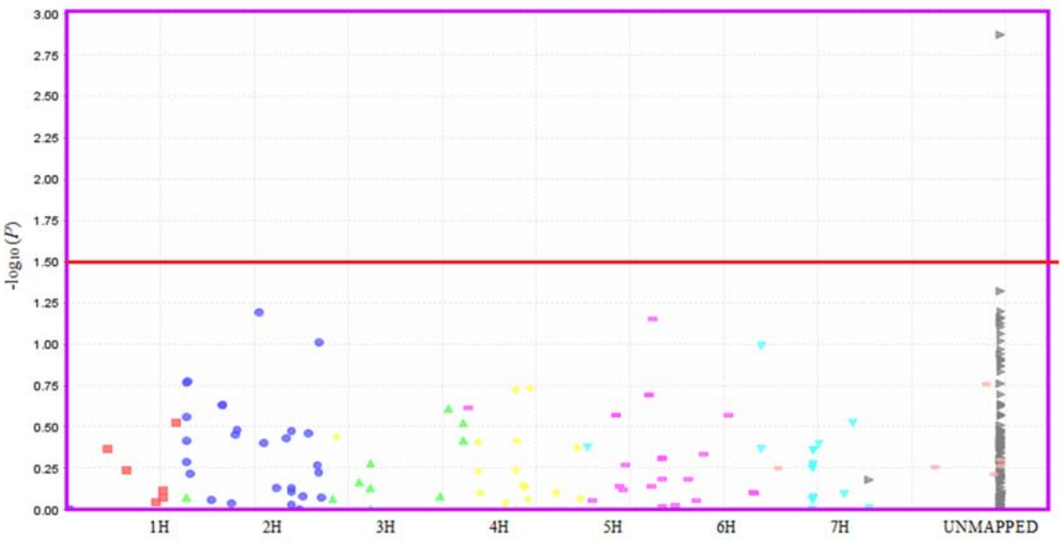

Manhattan Plot for ANTP in W1

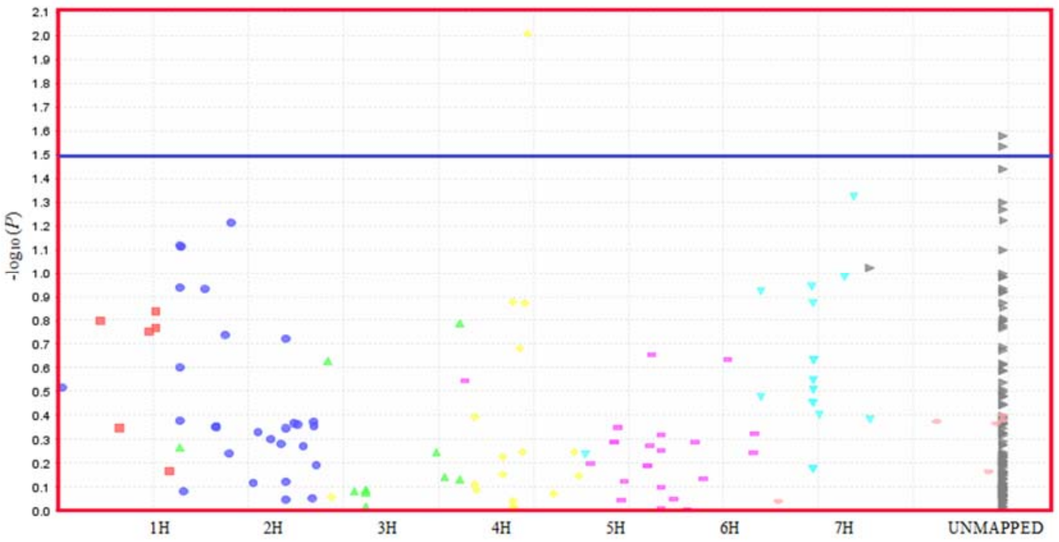

Manhattan Plot for ANTP in W2

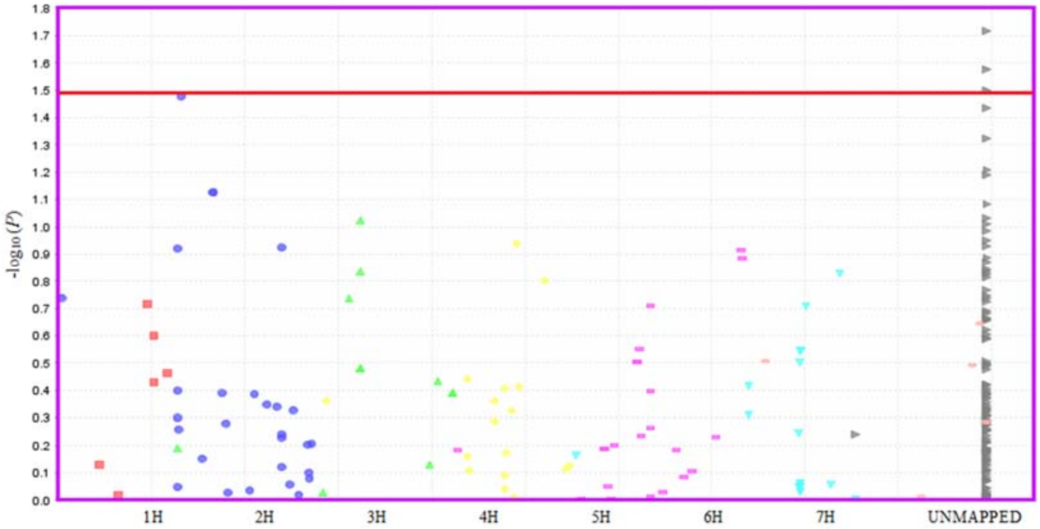

Manhattan Plot for FLL in W1

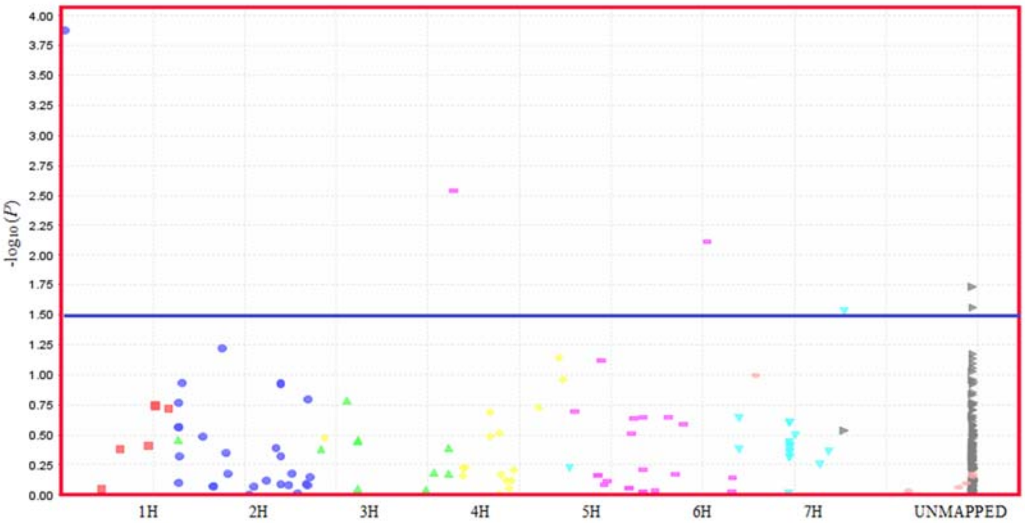

Manhattan Plot for FLL in W2

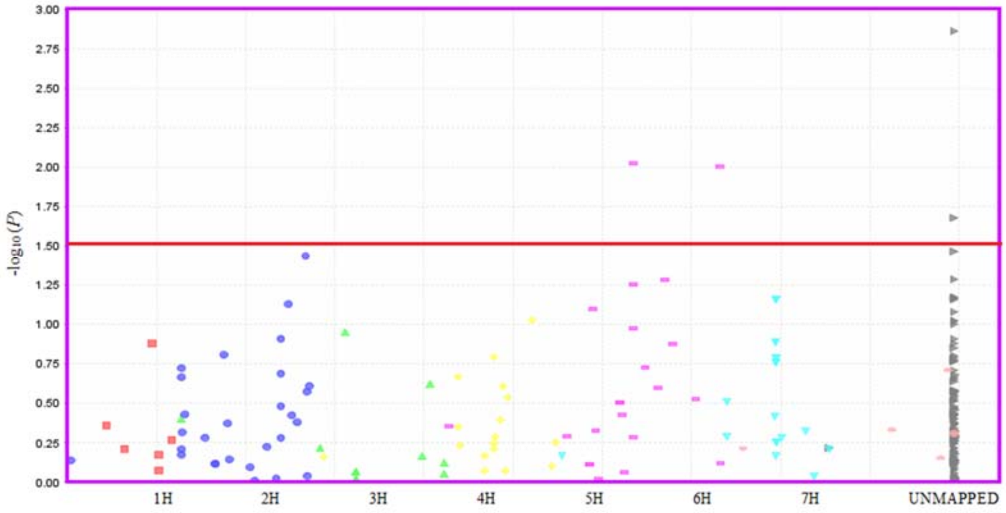

Manhattan Plot for FLSL in W1

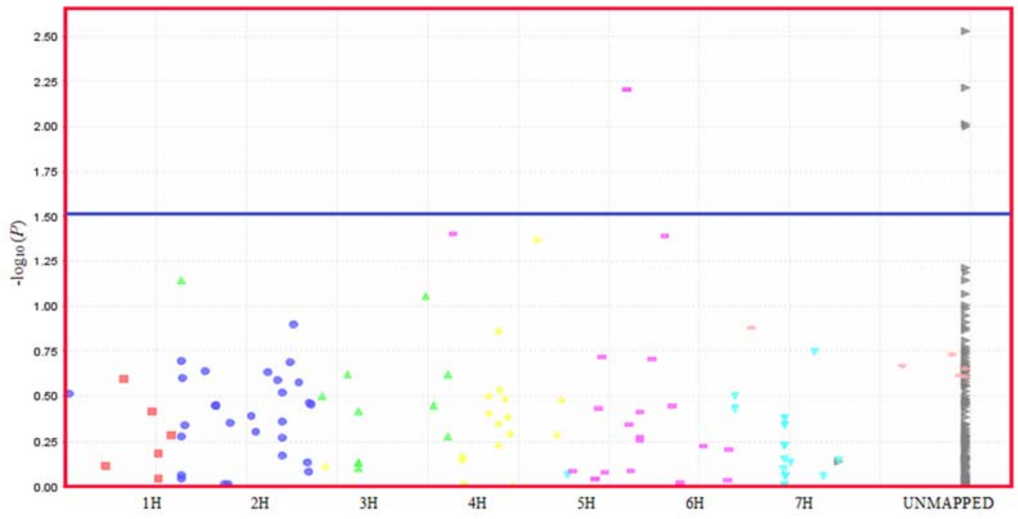

Manhattan Plot for FLSL in W2

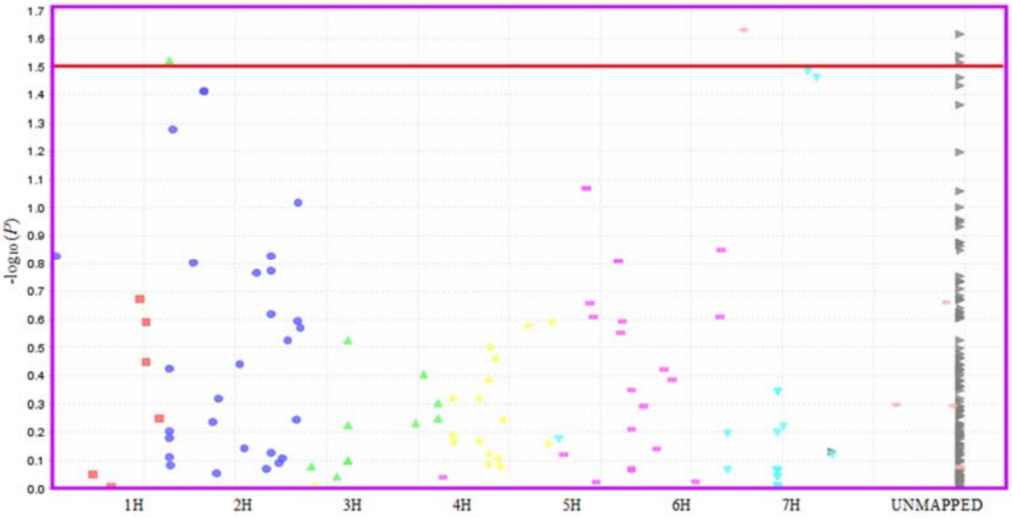

Manhattan Plot for FLW in W1

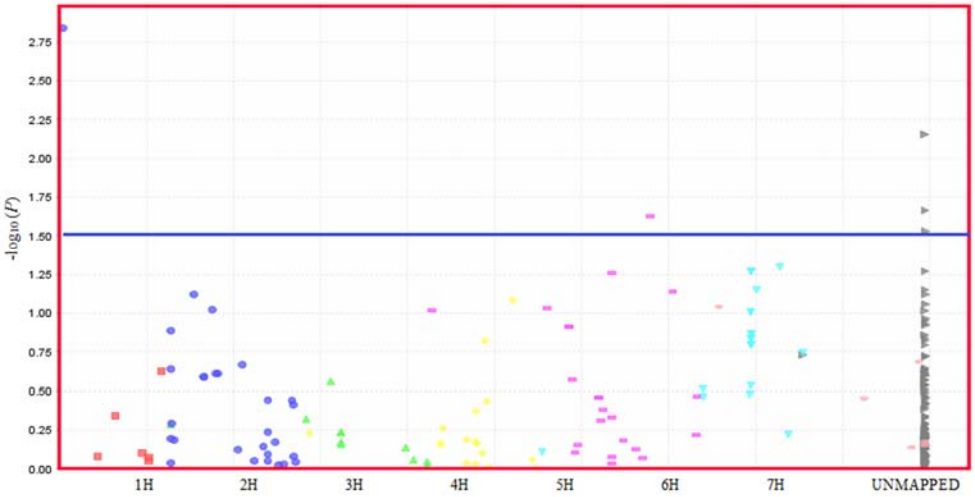

Manhattan Plot for FLW in W2

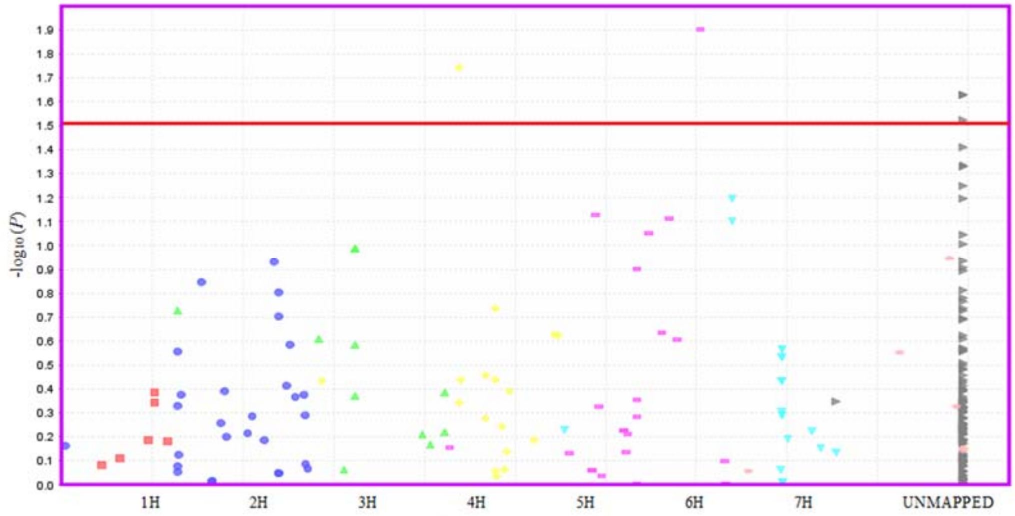

Manhattan Plot for IL in W1

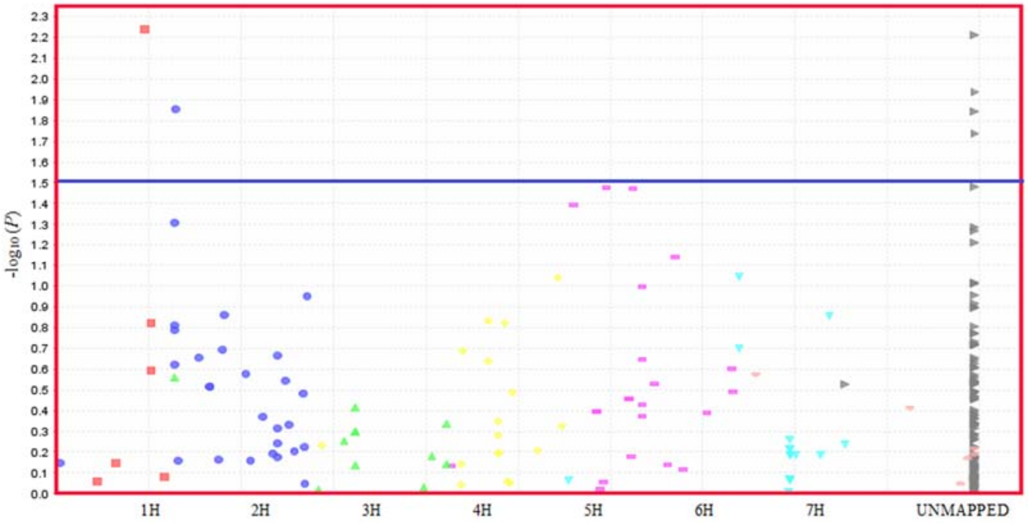

Manhattan Plot for IL in W2

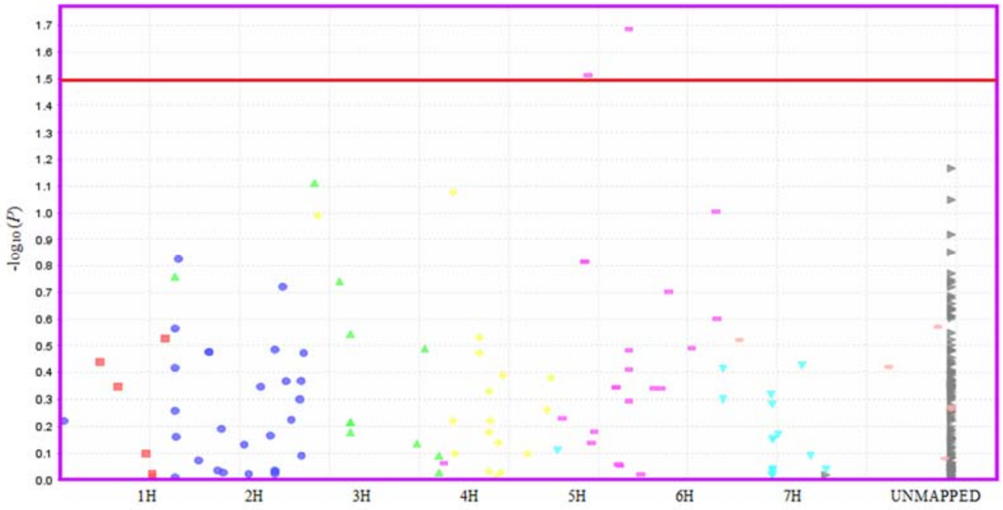

Manhattan Plot for MSL in W1

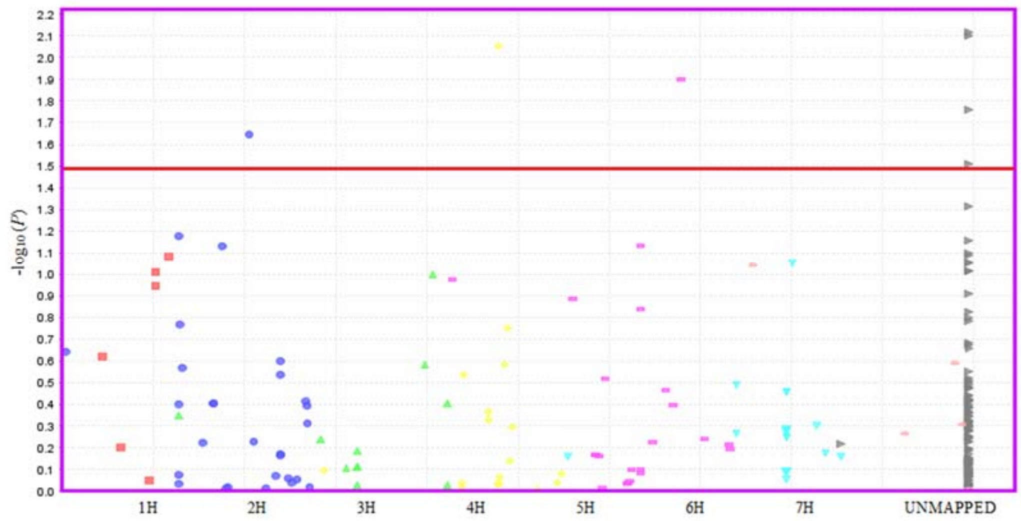

Manhattan Plot for MSN in W1

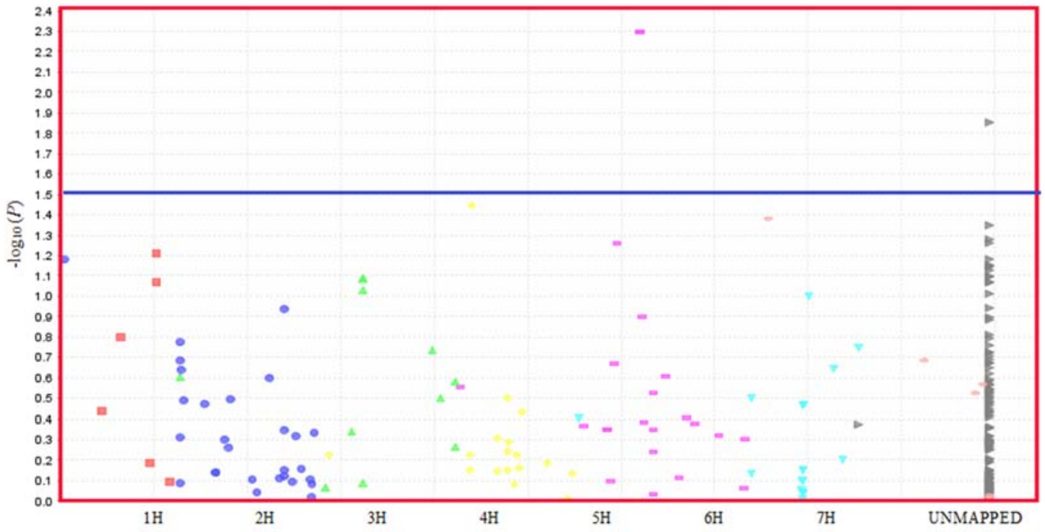

Manhattan Plot for MSN in W2

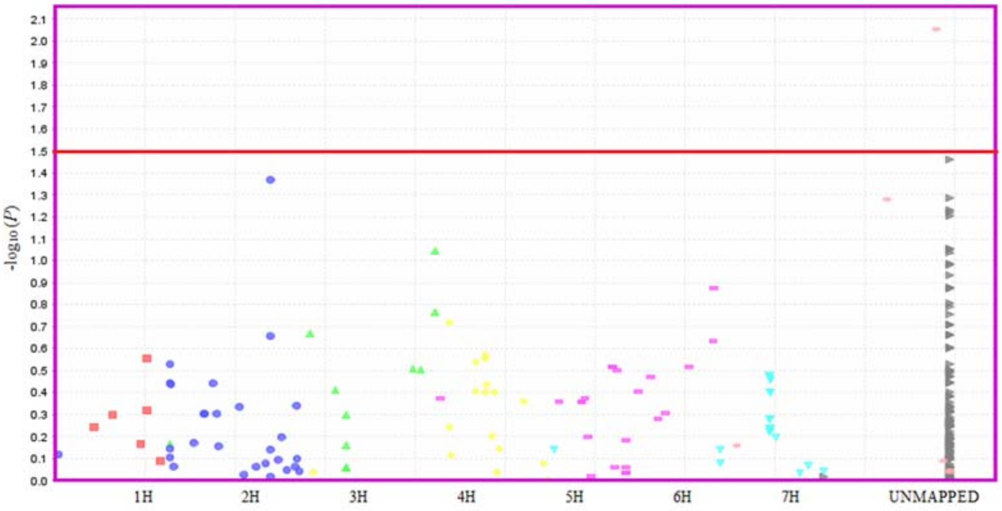

Manhattan Plot for PH in W1

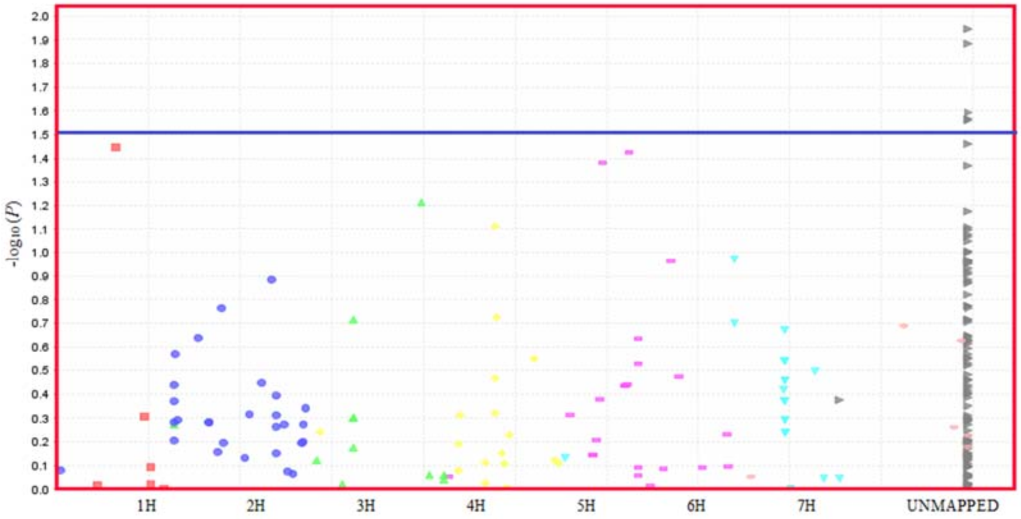

Manhattan Plot for PH in W2

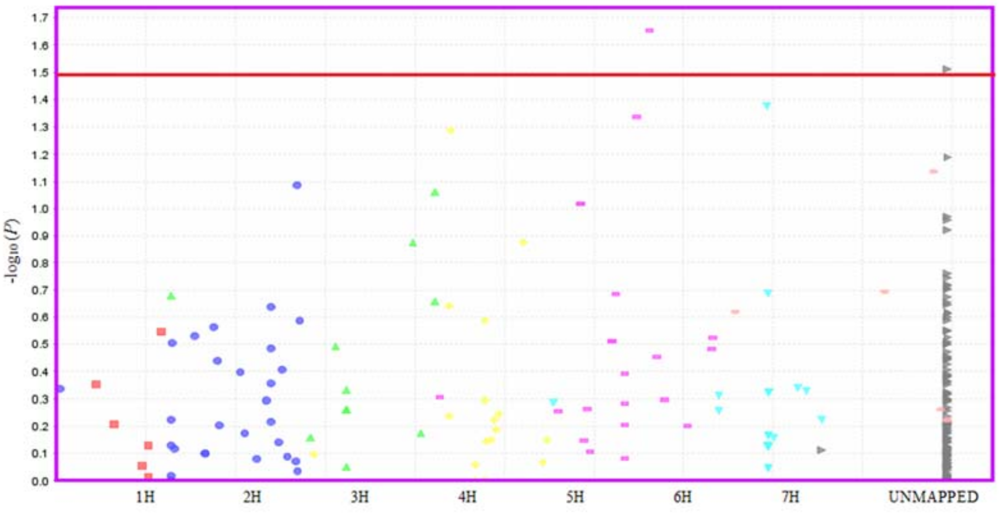

Manhattan Plot for PL in W1

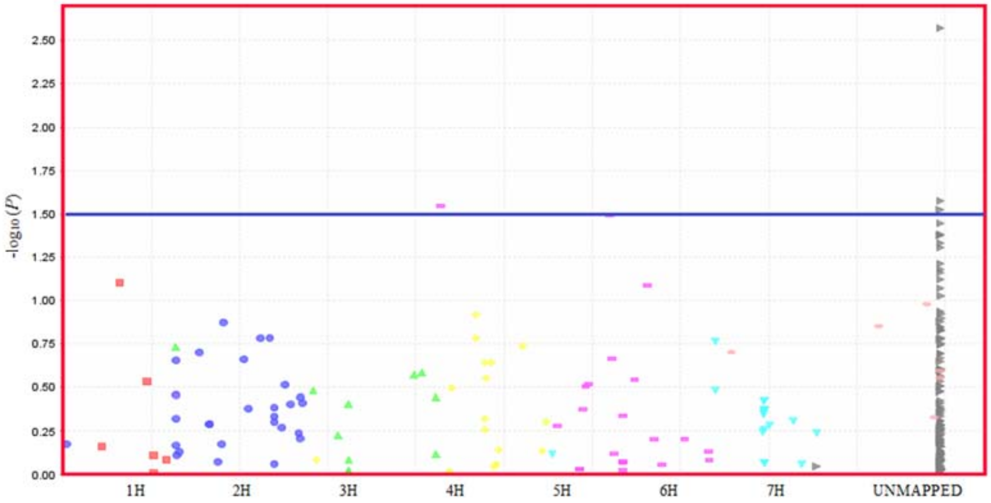

Manhattan Plot for PL in W2

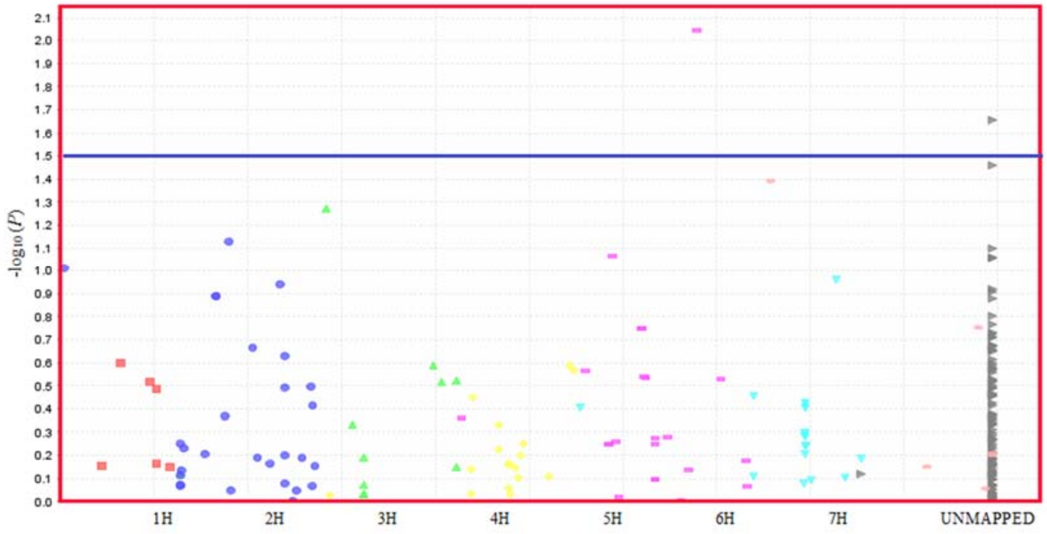

Manhattan Plot for GRS in W2
