# Supplementary material for: GWAS analysis in spring barley (Hordeum vulgare L.) for morphological traits exposed to drought
Source: PLoS One. 2018 Sep 27;13(9):e0204952. doi: 10.1371/journal.pone.0204952 (PMC6160164; doi:10.1371/journal.pone.0204952)
Supplement: S2 Fig — (PDF) [file pone.0204952.s002.pdf]

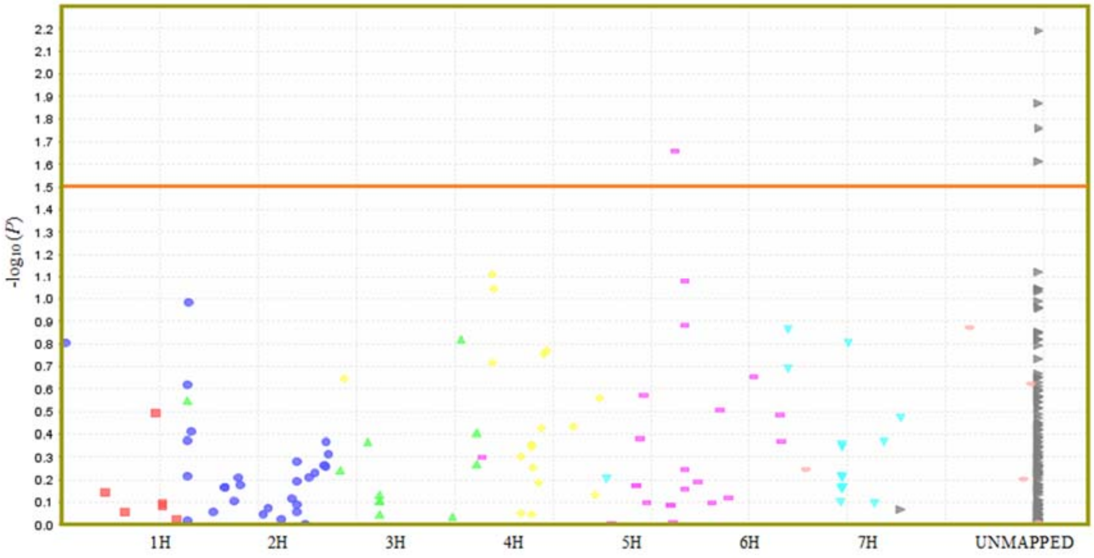

Manhattan plot for AL in D1

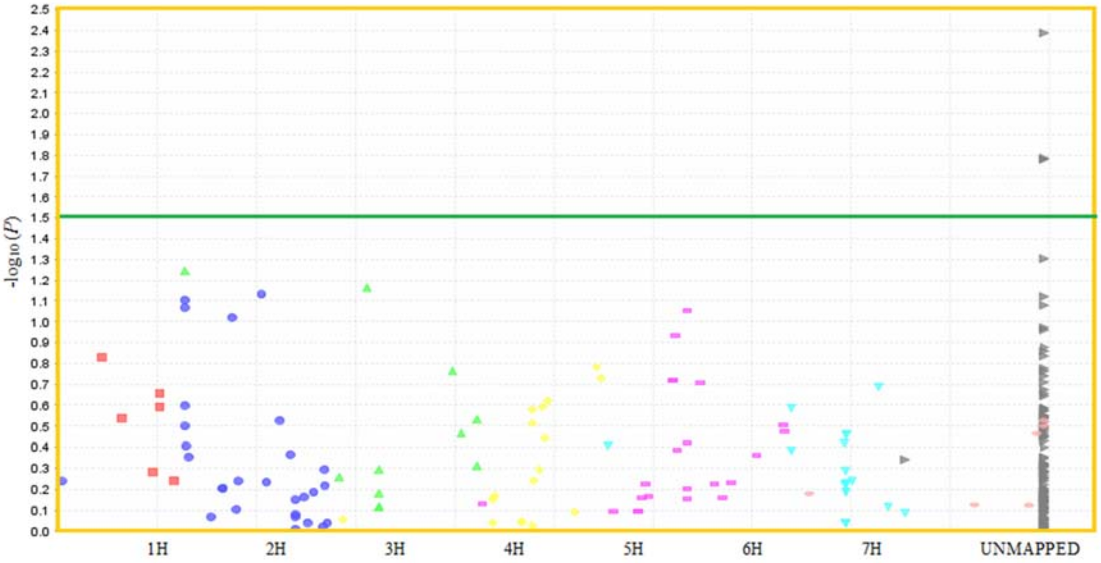

Manhattan plot for AL in D2

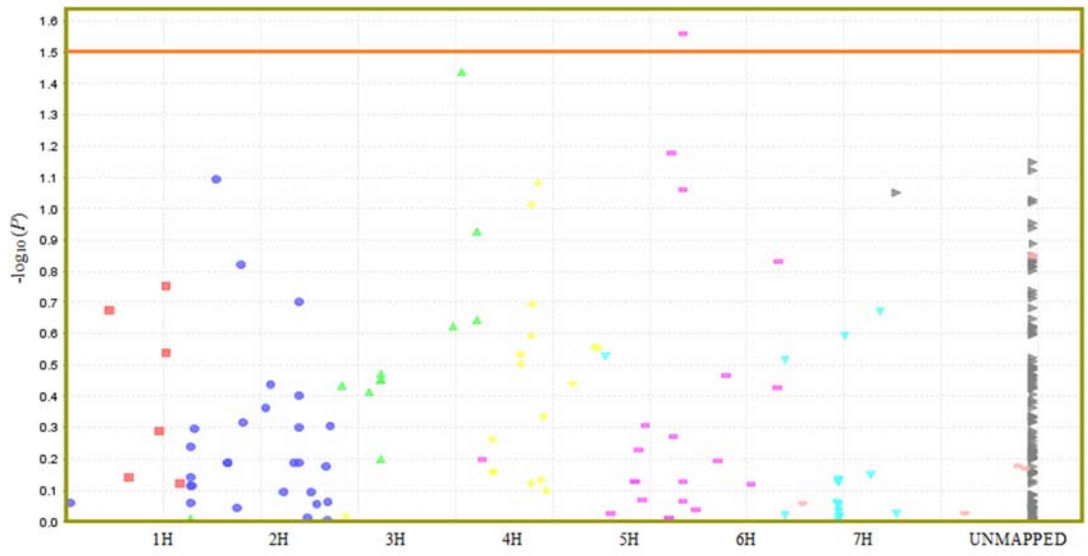

Manhattan plot for ANTP in D1

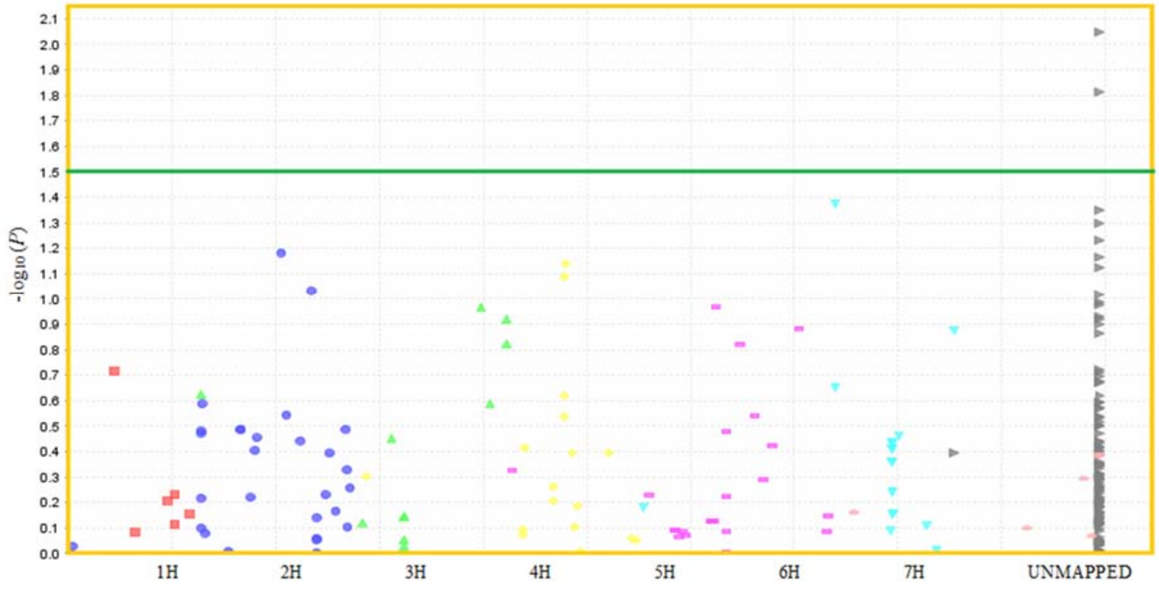

Manhattan plot for ANTP in D2

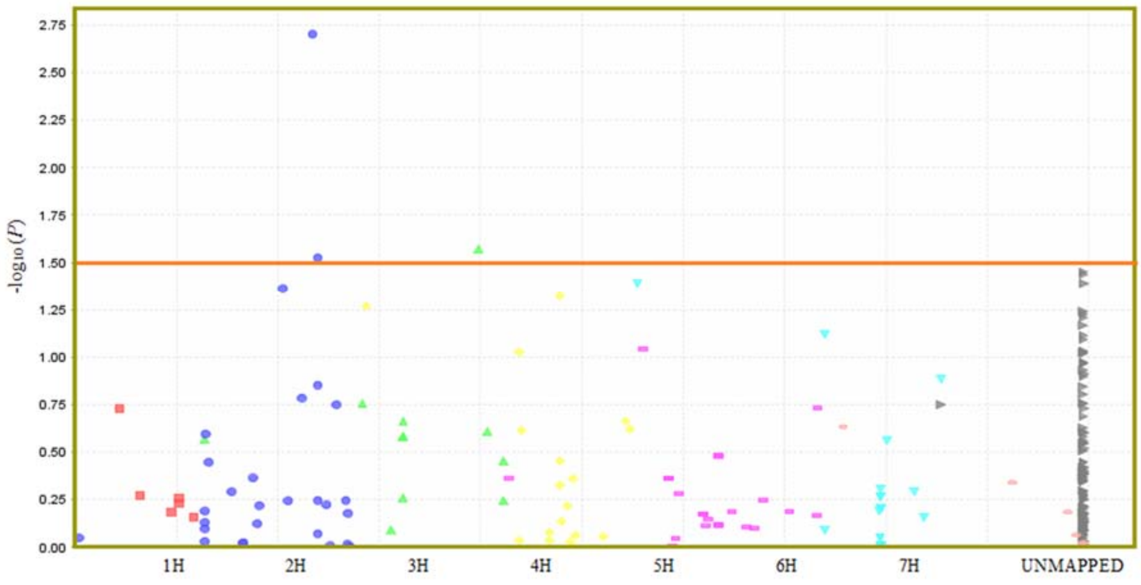

Manhattan plot for FLL in D1

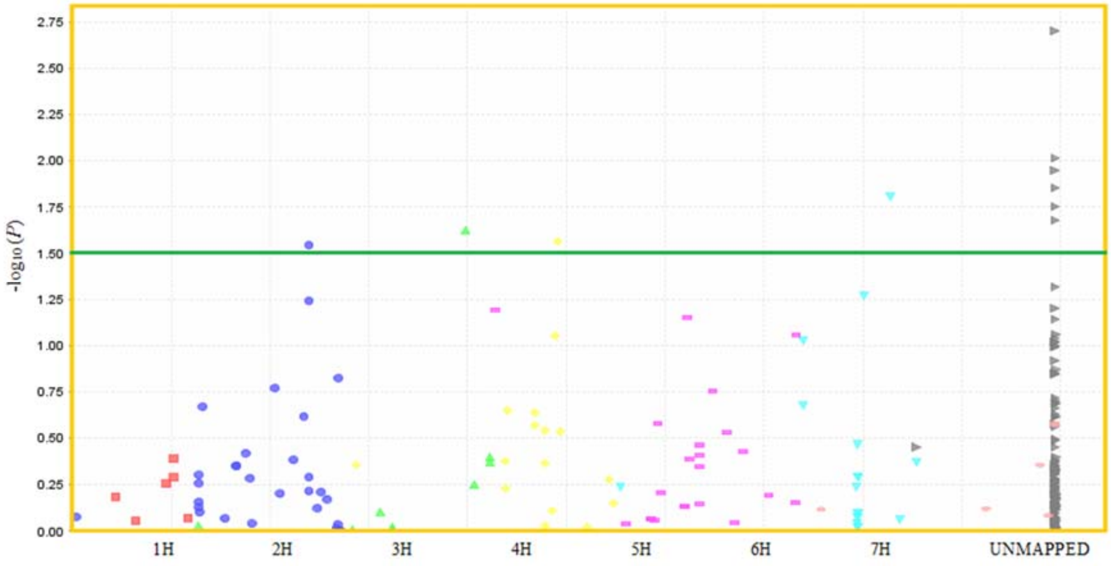

Manhattan plot for FLL in D2

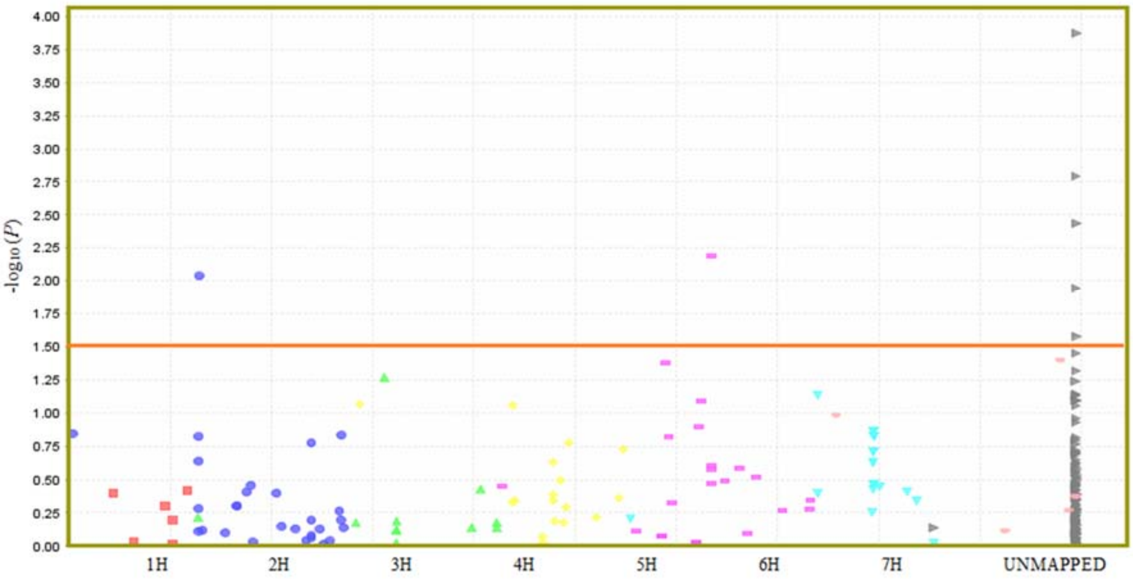

Manhattan plot for FLSL in D1

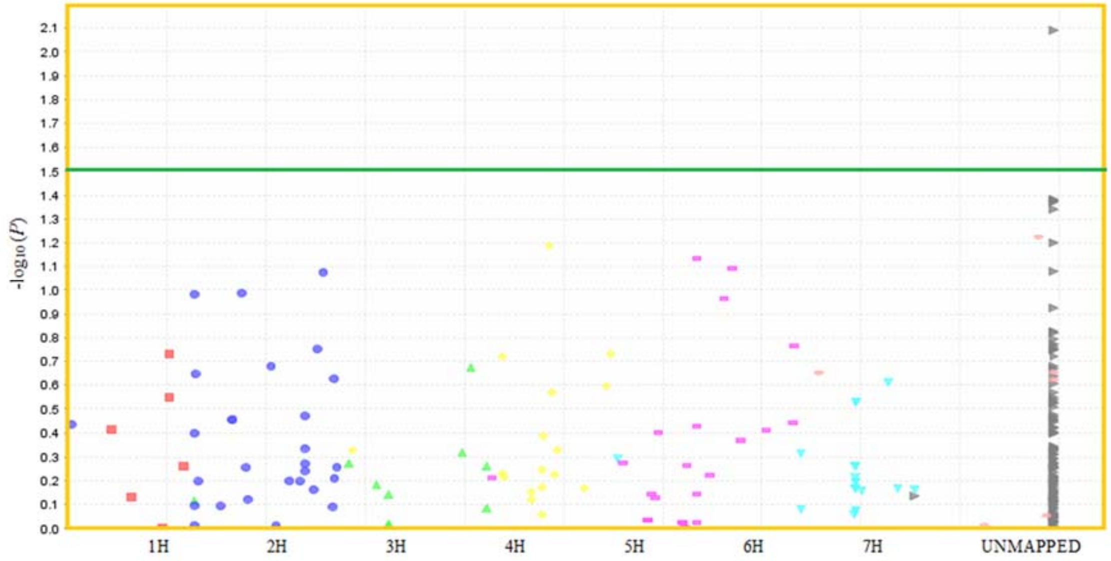

Manhattan plot for FLSL in D2

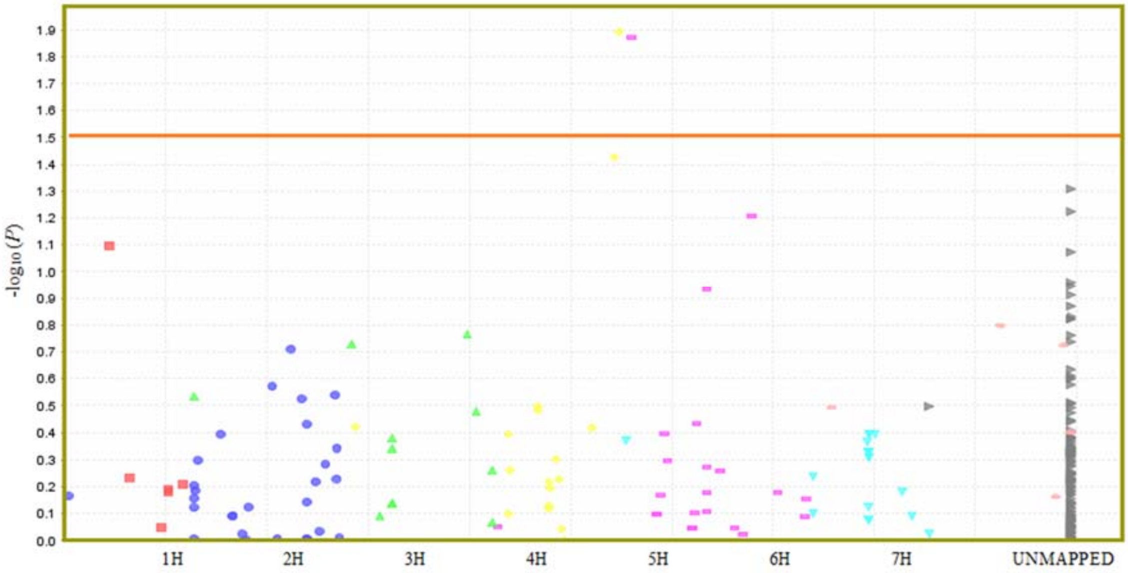

Manhattan plot for FLW in D1

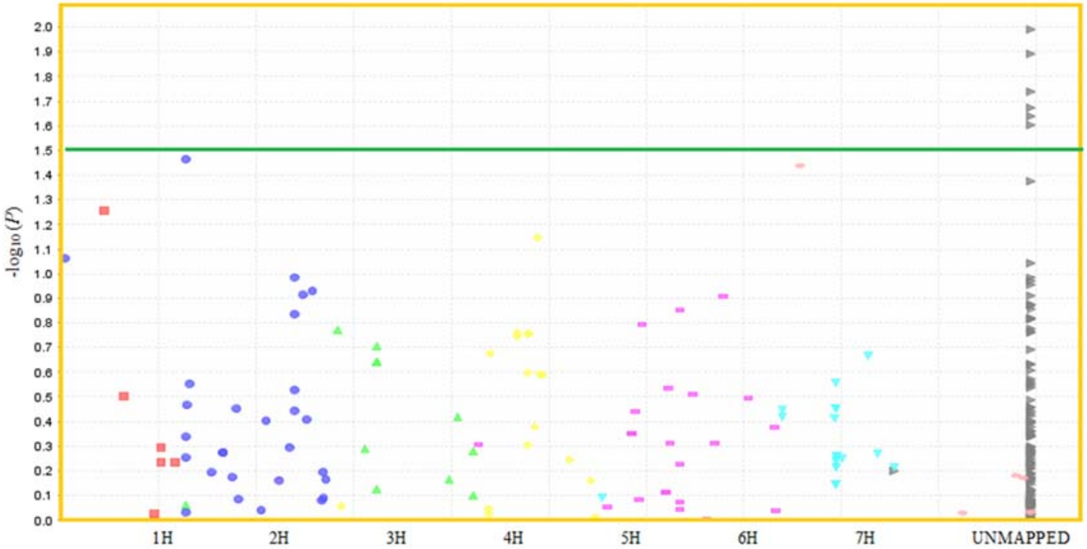

Manhattan plot for FLW in D2

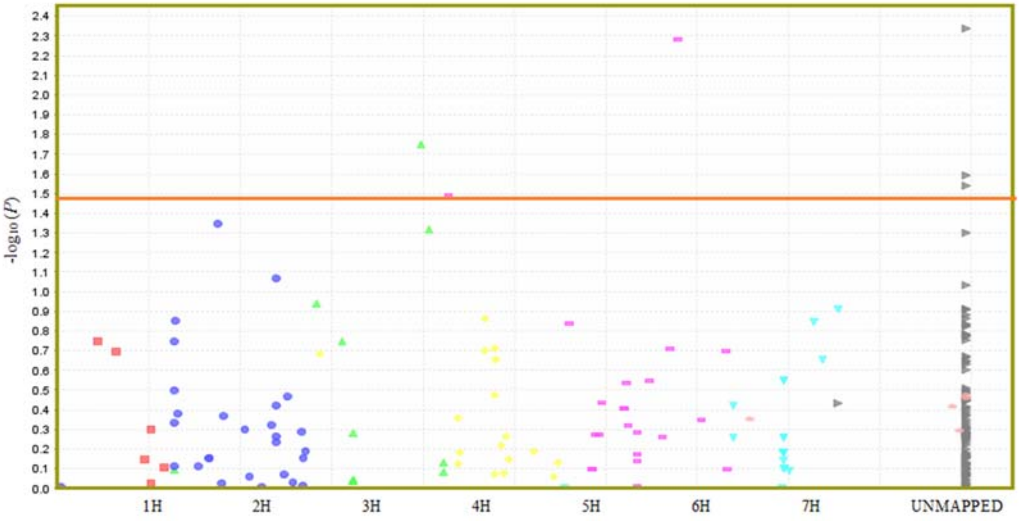

Manhattan plot for GRS in D1

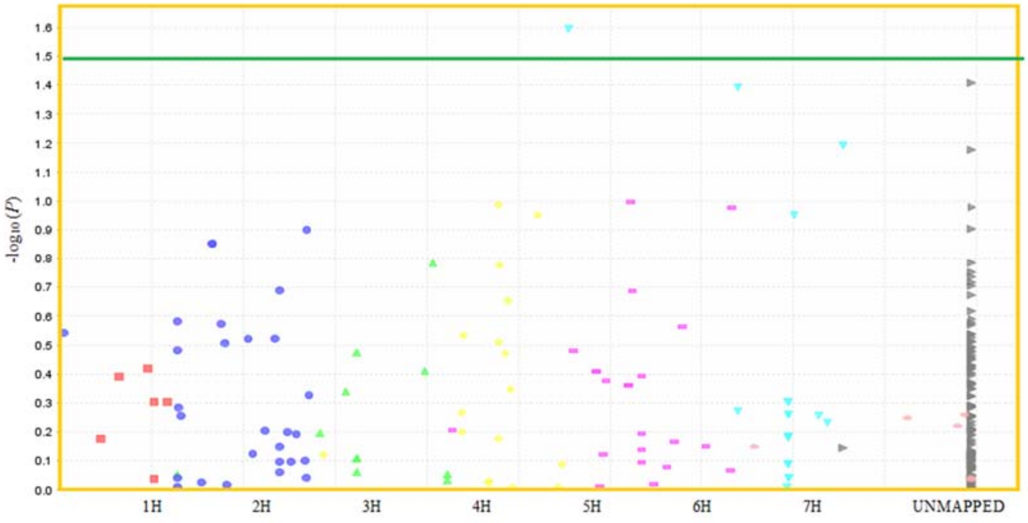

Manhattan plot for GRS in D2

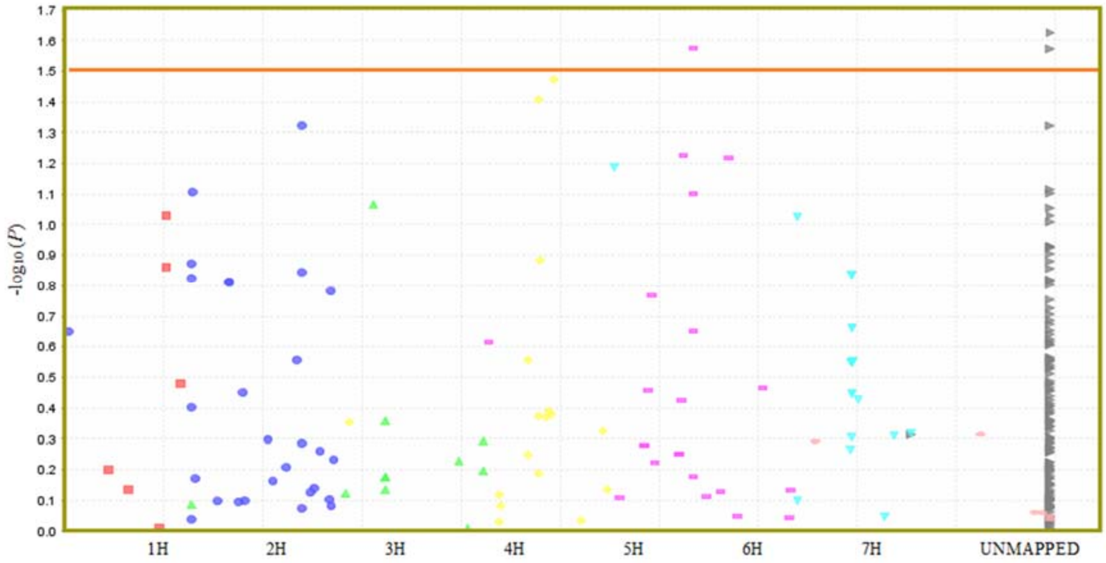

Manhattan plot for IL in D1

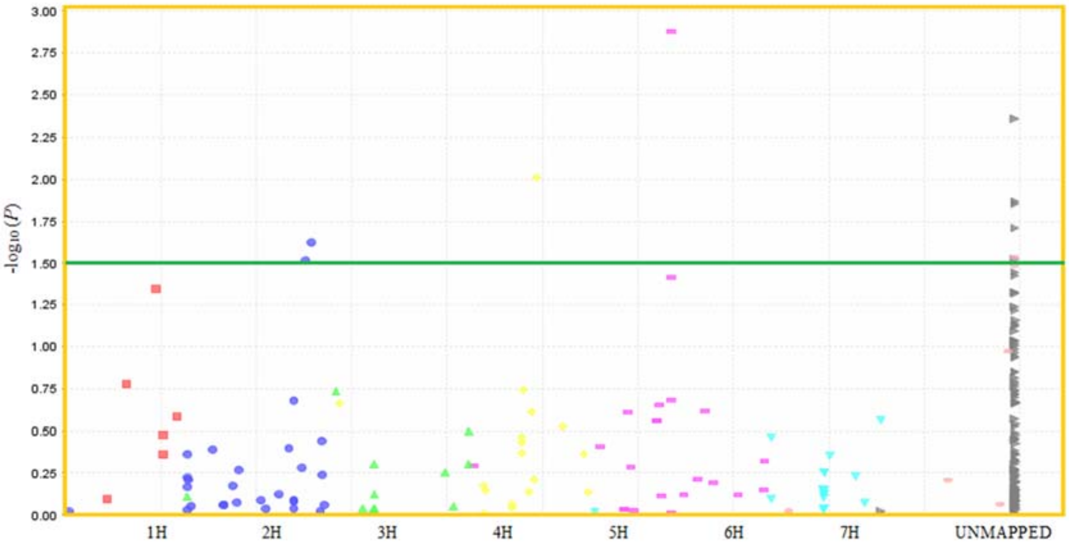

Manhattan plot for IL in D2

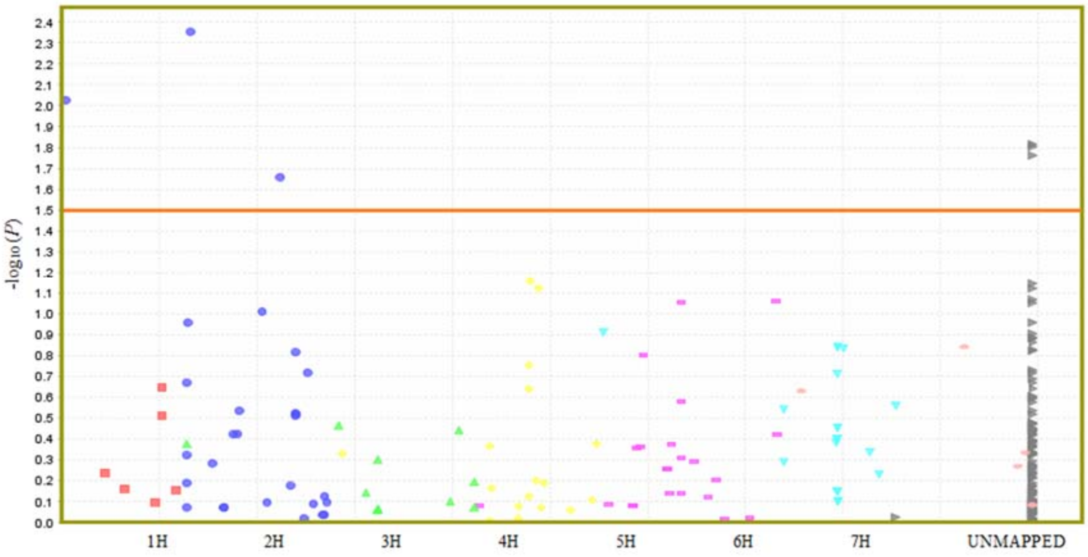

Manhattan plot for MSL in D1

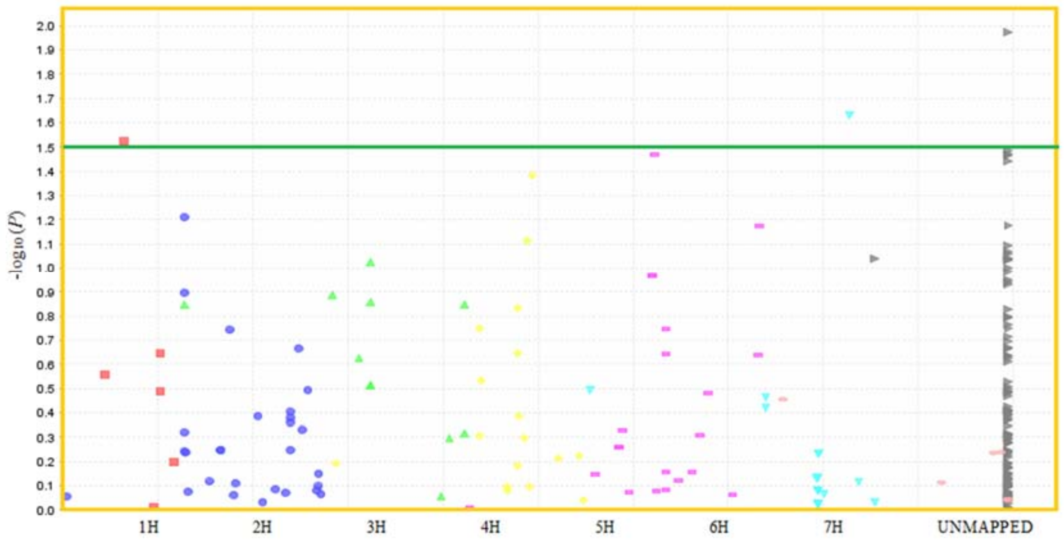

Manhattan plot for MSL in D2

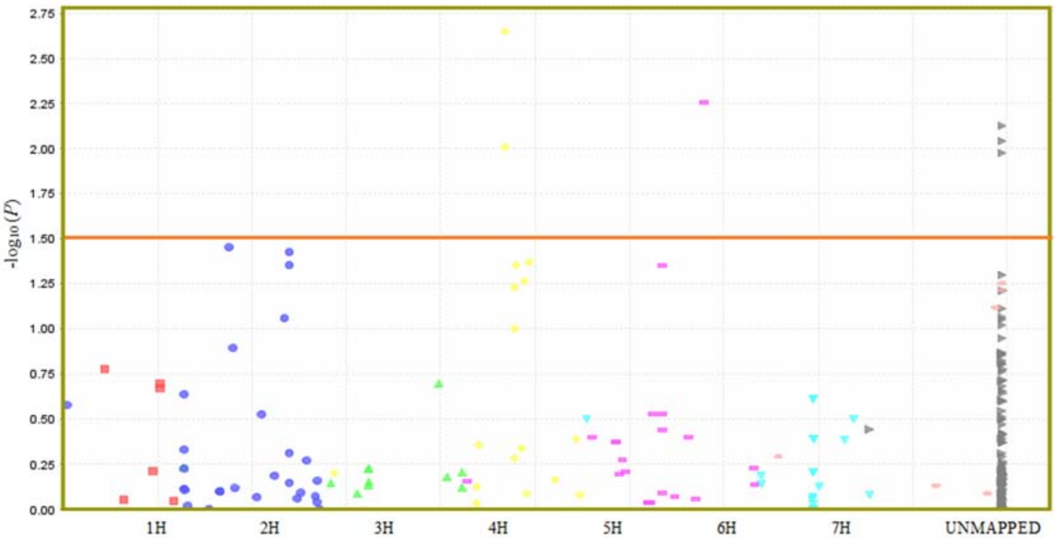

Manhattan plot for MSN in D1

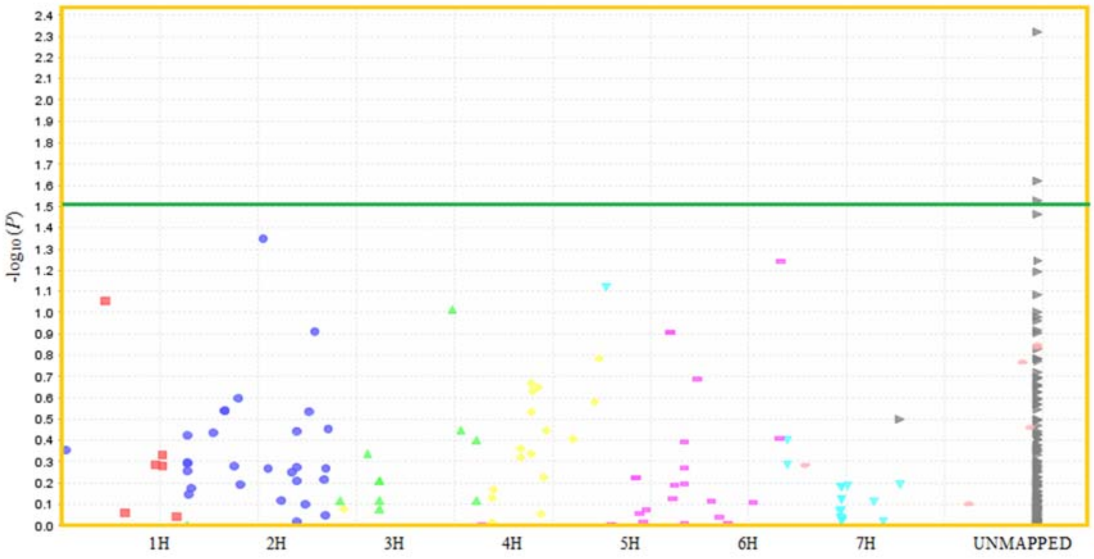

Manhattan plot for MSN in D2

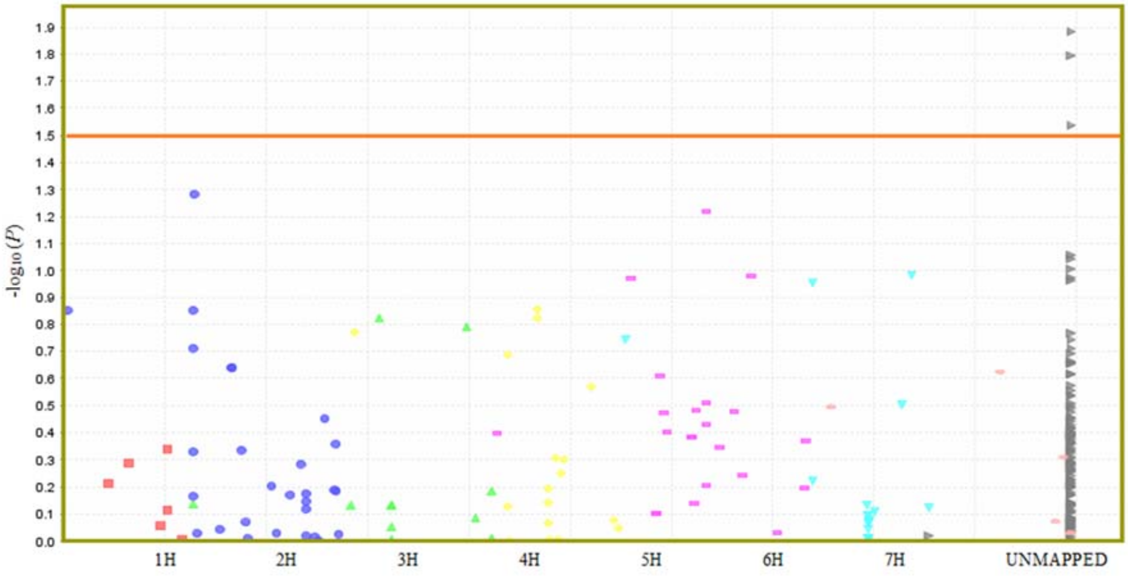

Manhattan plot for PH in D1

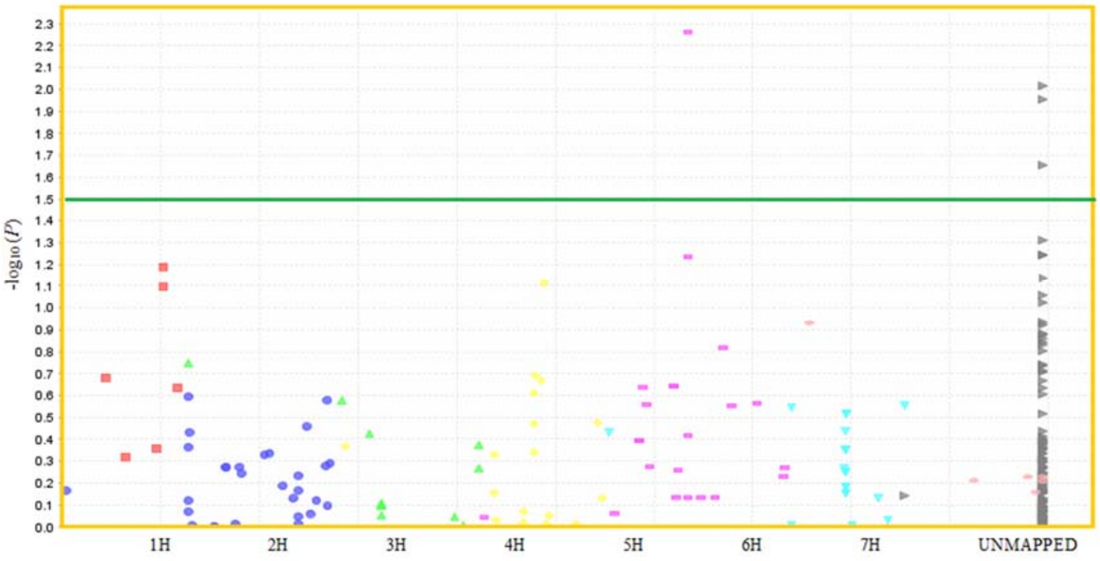

Manhattan plot for PH in D2

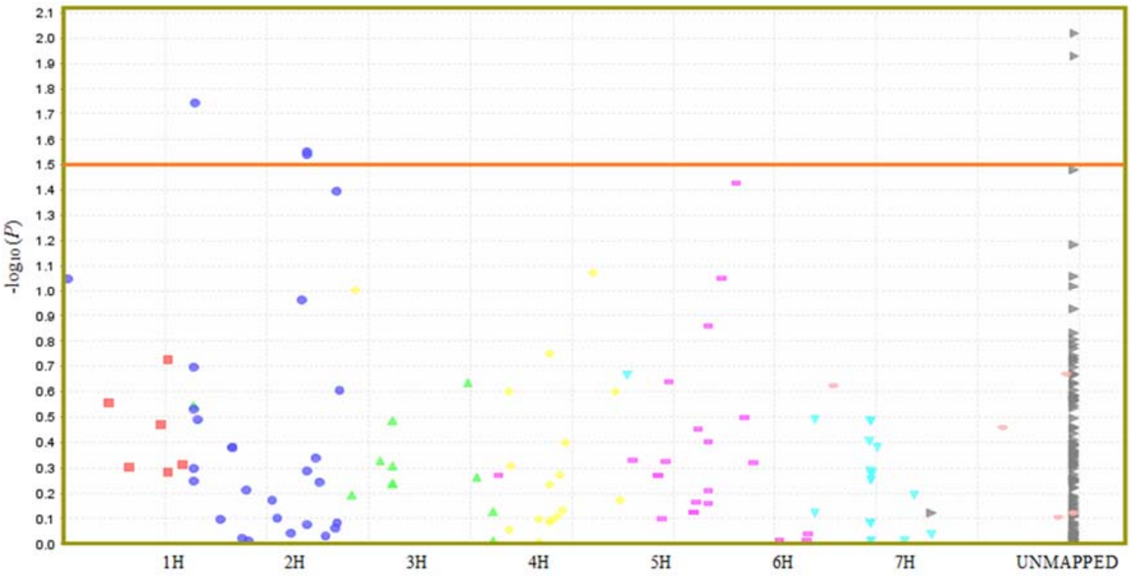

Manhattan plot for PL in D1

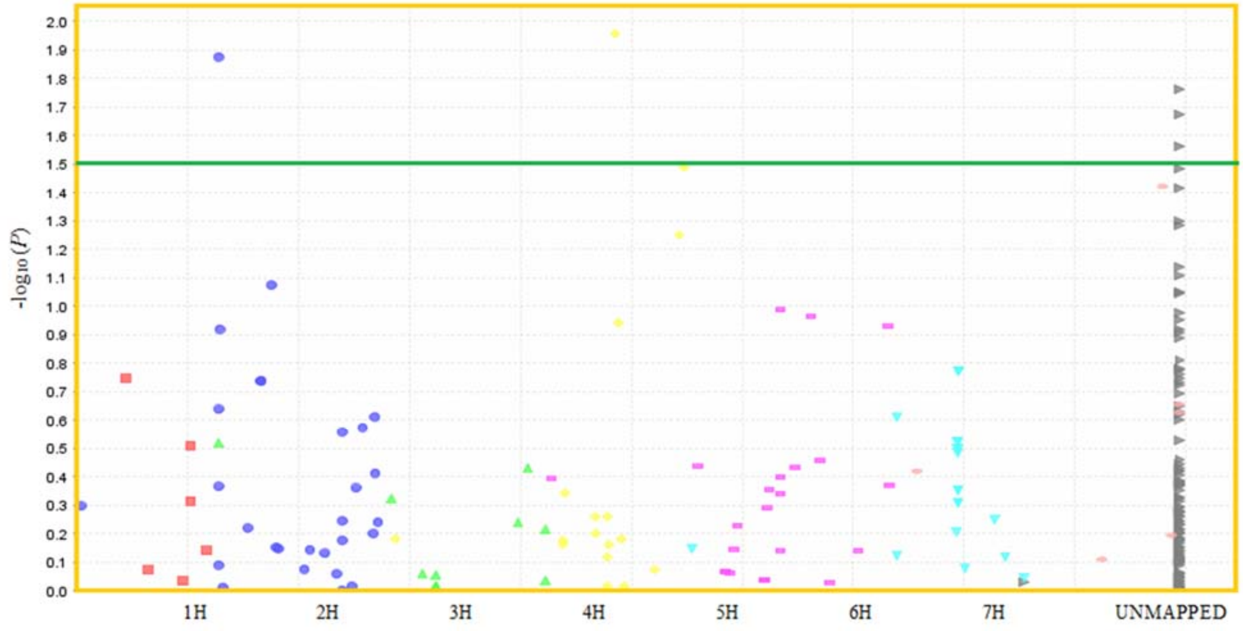

Manhattan plot for PL in D2
