# Supplementary material for: GWAS analysis in spring barley (Hordeum vulgare L.) for morphological traits exposed to drought
Source: PLoS One. 2018 Sep 27;13(9):e0204952. doi: 10.1371/journal.pone.0204952 (PMC6160164; doi:10.1371/journal.pone.0204952)
Supplement: S1 File — (PDF) [file pone.0204952.s003.pdf]

No.	cultivar	breeder
1	Abed4611	Abed Fonden
2	Abed5193	Abed Fonden
3	Abelone	New Farm Crops Ltd.
4	Adele	Plant Breeding International Ltd.
5	Akita	CPB
6	Alabama	Lochow-Petkus GmbH
7	Alanis	Sejet Planteforædling
8	Albright	Abed Fonden
9	Alexis	Saatzucht Josef Breun
10	Alliot	Pajbjergfonden
11	Annabell	Nordsaat Saatzucht GmbH
12	Annasofie	Pajbjergfonden
13	Apex	Cebeco Zaden BV
14	Aravis	Secobra Recherches
15	Ardila	Landbouwbureau Wiersum
16	Ariel	Svalöf Weibull AB
17	Aspen	Nickerson UK Ltd.
18	Astoria	Secobra Recherches
19	Barke	Saatzucht Josef Breun
20	Bartok	Nickerson UK Ltd.
21	Bella	Schweiger & Co.
22	Bereta	Abed Fonden
23	Blenheim	Plant Breeding International Ltd.
24	Bond	Sejet Planteforædling
25	Brenda	Saatzucht Hadmersleben GmbH
26	Brewster	New Farm Crops Ltd.
27	Brise	New Farm Crops Ltd.
28	Britta	Saatzucht Hadmersleben GmbH
29	Cadeau	Abed Fonden
30	Calypso	Carlsberg A/S
31	Camant	Carlsberg A/S
32	Caminant	Carlsberg A/S
33	Canut	Carlsberg A/S
34	Caruso	Carlsberg A/S
35	Caskant	Carlsberg A/S
36	Cathrine	Abed Fonden
37	Cecilia	Svalöf Weibull AB
38	Century	Nickerson UK Ltd.
39	Chalice	New Farm Crops Ltd.
40	Chamant	Carlsberg A/S
41	Chariot	Plant Breeding International Ltd.
42	Charlotte	Pflanzenzucht Dr. H.C. Carsten
43	Charon	Abed Fonden
44	Christian	Abed Fonden
45	Cicero	Sejet Planteforædling
46	Collie	Nickerson UK Ltd.
47	Cooper	New Farm Crops Ltd.
48	Cork	New Farm Crops Ltd.
49	Culma	Clovis Matton PVBA
50	Decanter	Nickerson Pflanzenzucht GmbH
51	Delibes	Nickerson UK Ltd.

52	Delita	Nordsaat Saatzucht GmbH
53	Derkado	Nordsaat Saatzucht GmbH
54	Dialog	Sejet Planteforædling
55	Digger	ICI Seeds Ltd.
56	Enigma	New Farm Crops Ltd.
57	Escort	Nickerson UK Ltd.
58	Etna	Abed Fonden
59	Evelyn	Saatzucht LFS
60	Extract	New Farm Crops Ltd.
61	Ferment	New Farm Crops Ltd.
62	Fusion	Sejet Planteforædling
63	Gant	Carlsberg A/S
64	Gesine	Nordsaat Saatzucht GmbH
65	Give	Abed Fonden
66	Goldie	Svalöf Weibull AB
67	Hanka	Saatzucht Hadmersleben GmbH
68	Henni	Nordsaat Saatzucht GmbH
69	Heron	ICI Seeds Ltd.
70	Hydrogen	Pajbjergfonden
71	Jacinta	Pajbjergfonden
72	Jarek	Agricultural Research Institute Kromeriz, Ltd.
73	Jersey	Cebeco Zaden BV
74	Jill	Svalöf Weibull AB
75	Korinna	Saatzucht Hadmersleben GmbH
76	Krona	Saatzucht Hadmersleben GmbH
77	Lamba	Sejet Planteforædling
78	Lenka	Saatzucht Hadmersleben GmbH
79	Libelle	Saatzucht Josef Breun
80	Limbo	Svalöf Weibull AB
81	Linus	Svalöf Weibull AB
82	Lisbet	Pajbjergfonden
83	Loma	Abed Fonden
84	Lux	Sejet Planteforædling
85	Lysiba	Sejet Planteforædling
86	Lysimax	Sejet Planteforædling
87	Madonna	Lochow-Petkus GmbH
88	Madras	Lochow-Petkus GmbH
89	Mandolin	Advanta Seeds BV
90	Maresi	Lochow-Petkus GmbH
91	Marina	Lochow-Petkus GmbH
92	Maud	Svalöf Weibull AB
93	Meltan	Svalöf Weibull AB
94	Mentor	Svalöf Weibull AB
95	Merete	Pajbjergfonden
96	Mie	Svalöf Weibull AB
97	Miralix	Sejet Planteforædling
98	Neruda	Nickerson UK Ltd.
99	Nevada	Secobra Recherches
100	Nizza	Saatzucht Josef Breun
101	Odin	Plant Breeding International Ltd.
102	Optic	New Farm Crops Ltd.
103	Optima	Saatzucht Josef Breun

104	Orthega	Lochow-Petkus GmbH
105	Otira	Sejet Planteforædling
106	Paloma	Abed Fonden
107	Pasadena	Lochow-Petkus GmbH
108	Peel	New Farm Crops Ltd.
109	Peggy	Saatzuchtgesellschaft Streng's Erben GmbH & Co. KG
110	PF11011-52	Pajbjergfonden
111	PF11202-53	Pajbjergfonden
112	Polygena	Saatzucht Hadmersleben GmbH
113	Pongo	Svalöf Weibull AB
114	Potter	Svalöf Weibull AB
115	Prestige	Plant Breeding International Ltd.
116	Princesse	Nordsaat Saatzucht GmbH
117	Prisma	Landbouwbureau Wiersum
118	Prolog	Sejet Planteforædling
119	Prominant	Sejet Planteforædling
120	Punto	Sejet Planteforædling
121	Ragtime	Advanta Seeds BV
122	Reggae	Advanta Seeds BV
123	Ricarda	Nickerson UK Ltd.
124	Riga	Abed Fonden
125	Roxana	Saatzucht Josef Breun
126	Sabel	New Farm Crops Ltd.
127	Saloon	New Farm Crops Ltd.
128	Scarlett	Saatzucht Josef Breun
129	Senor	Sejet Planteforædling
130	Shamu	Sejet Planteforædling
131	SJ5095	Sejet Planteforædling
132	Static	New Farm Crops Ltd.
133	Steffi	Ackerman & Co, Saatzucht Irlbach
134	Sultane	Secobra Recherches
135	Teal	New Farm Crops Ltd.
136	Texane	Secobra Recherches
137	Thuringia	Ackerman & Co, Saatzucht Irlbach
138	Tirup	Svalöf Weibull AB
139	Tofta	Svalöf Weibull AB
140	Trebon	Svalöf Weibull AB
141	Tremois	Verneuil Semences
142	Trianon	Secobra Recherches
143	UN AE 3.1	Unisigma, GIE Recherche et Sélection
144	Vada	IVP Wageningen
145	Verona	Abed Fonden
146	Vintage	New Farm Crops Ltd.
147	Viskosa	Nordsaat Saatzucht GmbH
148	Wren	Advanta/Zeneca
