# Supplementary material for: GWAS analysis in spring barley (Hordeum vulgare L.) for morphological traits exposed to drought
Source: PLoS One. 2018 Sep 27;13(9):e0204952. doi: 10.1371/journal.pone.0204952 (PMC6160164; doi:10.1371/journal.pone.0204952)
Supplement: S2 File — (PDF) [file pone.0204952.s004.pdf]

Table S2- Variance analysis for all traits, sum of squares was shown.

Source	ANTP	PH	IL	MSL	AL	FLSL	PL	FLL	FLW	MSN	GRS
Genotype	410.88**	542.41**	1444.88**	1044.96**	987.24**	1147.44**	1019.12**	1131.62**	1644.85**	570.92**	2742.79**
Year	150.61**	44.59**	104.96**	0.001	16.78**	0.02	147.43**	184.45**	60.49**	5.22**	5.89**
Environment	53.05**	56.20**	244.69**	5.08	15.09**	129.24**	316.83**	1.79**	2.10	3.45	60.21**
Genotype × Year	456.10**	223.88**	913.83**	513.60**	552.60**	553.20**	664.43**	723.40**	743.51**	362.04**	560.56**
Genotype × Environment	368.27**	182.77	659.03**	365.54**	507.73**	457.58**	471.34**	565.81**	807.80**	230.18**	392.41**
Year × Environment	28.15**	13.34**	42.27**	0.91	13.55**	36.56**	154.29**	0.97	0.001	0.06	8.70**
Genotype × Year × Environment	377.68**	223.91**	793.87**	439.48**	437.23**	460.87**	377.87**	630.16**	707.79**	225.51**	449.43**

**: Significant at 0.01 level
